# A dsRNA Viral Transcriptional Regulator Evades Innate Immunity by Hijacking Host CoTranscription Factor DHX9

**DOI:** 10.1002/advs.202512262

**Published:** 2025-12-22

**Authors:** Xueyang Pang, Shiyu Liu, Yixiao Zhu, Meleana M. Hinchman, Nan Qiao, Xuejun Li, Weiqian Dai, Yingying Tong, Man Xing, Jiling Ren, John S. L. Parker, Yingying Guo, Dongming Zhou

**Affiliations:** ^1^ Department of Pathogen Biology School of Basic Medical Sciences Tianjin Medical University Tianjin China; ^2^ National Clinical Research Centre for Geriatric Disorders Xiangya Hospital Central South University Changsha Hunan China; ^3^ Baker Institute for Animal Health College of Veterinary Medicine Cornell University Ithaca New York USA; ^4^ Cornell Institute of Host‐Microbe Interactions and Disease Cornell University Ithaca New York USA

**Keywords:** DHX9, NF‐kB, reovirus, R‐loop, transcription

## Abstract

Viral transcriptional regulators (vTRs) have emerged as potent factors that shape the host anti‐viral gene expression programs. Delineating the molecular mechanisms by which vTRs inhibit or promote transcription will provide fundamental insights for developing anti‐viral strategies. Using Mammalian orthoreovirus (REOV) as a model system, a new viral mechanism by which viruses antagonize the host innate immune response is identified. We found that the REOV outer capsid protein σ3 functions as a vTR to suppress NF‐κB gene expression via its direct interaction with the host helicase DHX9. Mechanistically, σ3 impairs the initial recruitment of Pol II by disrupting the interaction between DHX9 and Pol II. More interestingly, σ3 suppresses DHX9 helicase activity, resulting in the aberrant accumulation of R‐loops at promoter‐proximal regions, thereby affecting Pol II pause‐release and ultimately suppressing NF‐κB gene expression. Together, these findings reveal an unprecedented strategy employed by a viral protein that regulates anti‐viral gene expression by directly modulating the host transcription factor DHX9.

## Graphical abstract

This study identifies a new viral mechanism by a viral protein σ3 that functions as a vTR to suppress NF‐κB gene expression via its direct interaction with the host helicase DHX9. Through their interaction, σ3 not only impairs the initial recruitment of Pol II but also affects Pol II pause‐release and ultimately suppresses NF‐κB gene expression.

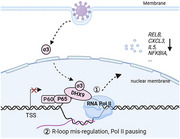

## 1 Introduction

To successfully infect hosts, viruses encode various proteins that help them overcome the host's immune responses. Among these proteins, viral transcription regulators (vTRs) play essential roles, by inhibiting viral sensing, modulating immune signaling, and regulating cell cycle progression and metabolism [1, 2]. Over 400 vTRs have been identified across 20 DNA and RNA virus families [3]. By precisely controlling the temporal expression of viral or host genes, vTRs help to create an environment beneficial for productive infection, making them attractive therapeutic targets [4]. Despite the essential roles of vTRs in virus‐host warfare, our understanding of their molecular functions remains largely unknown.

The mechanisms by which vTRs modulate host gene expression include: a) direct interactions with the host genome by binding DNA (e.g., Zta from EBV binds the host genome through its basic leucine zipper domain) [5]; b) interactions with host transcription factors (e.g., EBNA2 from EBV interacts with human transcription factor to occupy the host genome) [6]; c) targeting the transcriptional machinery (e.g., Tat from HIV precisely controls RNA polymerase II recruitment and pause release to fine‐tune the target genes) [7]; and d) modification of chromatin (e.g., HBx from HBV mediates epigenetic modification of host chromatin through interacting with DNMT3A and HDAC1) [8]. The remarkable structural diversity of vTRs poses a significant challenge in predicting their mechanism based on structure [9]. Current research on vTRs relies on individual case studies, limiting our understanding of the functions of vTRs.

Eukaryotic transcription is regulated at multiple levels [10, 11]. One mechanism occurs through the formation of R‐loops during transcription. Physiological R‐loops may form in *cis* as a transcriptional byproduct when the newly transcribed RNA strand hybridizes with the complementary single‐stranded DNA template, leading to the displacement of the non‐template DNA strand. Increasing evidence suggests that R‐loops also form in *trans* independently of the transcriptional site [12, 13]. Regulation of transcription by forming R‐loops is complex and controversial, as their formation can either be detrimental or beneficial to transcription [12, 14, 15]. In general, the formation of R‐loops during transcription is considered harmful, as they structurally impede continued transcription. However, R‐loop formation may facilitate a variety of DNA‐related activities, including transcription termination and gene regulation, telomere stability, and DNA repair [14, 16, 17]. The dual nature of R‐loops implies that their formation, localization, and removal are strictly controlled. Despite significant progress in understanding the regulation and functions of R‐loops under pathological conditions such as DNA damage, genomic instability, neurodegenerative diseases, and tumorigenesis, studies on the interplay between R‐loop formation and virus infection remain limited.

Mammalian orthoreoviruses (REOV) are non‐enveloped viruses with a ten‐segment, double‐stranded RNA genome. REOV infects a variety of mammals including humans. In humans, infection early in life has been linked to the development of celiac disease, but for the most part REOV causes minimal disease. Because of this some REOV strains are being developed as oncolytic viruses [18, 19]. REOV infection activates multiple signaling pathways in cells. The Nuclear factor kappa‐light‐chain enhancer of B‐cells (NF‐κB) family of host transcription factors regulates immune responses, inflammation, cell survival and development [20]. NF‐κB consists of homodimers or heterodimers of five different subunits. Anti‐viral signaling via the p65/p50 heterodimeric form of NF‐κB is central to the innate immune response to viral infection and leads to the expression of hundreds of anti‐viral genes. The regulation of the NF‐κB pathway during REOV infection is a complex process. Early in infection, NF‐κB is activated; however, later in infection, NF‐κB‐dependent gene expression activity is suppressed, and exogenous NF‐κB agonists, such as tumor necrosis factor‐alpha (TNF‐α), fail to activate the NF‐κB pathway [21, 22]. The REOV gene product, σ3, is sufficient to inhibit the expression of NF‐κB‐dependent genes activated by TNF‐α or viral genomic dsRNA [21]. The mechanism by which σ3 inhibits NF‐κB transcription does not involve inhibition of nuclear entry of p65. In infected cells, σ3 localizes to both the cytoplasmic viral factories and the nucleus [23]. σ3 directly inhibits PKR activation and stress granule formation in the cytoplasm [24], however, the nuclear function of σ3 is still unknown. These observations suggest that σ3 may directly regulate transcription within the nucleus.

To further investigate how σ3 regulates the transcription of NF‐κB‐dependent genes, we employed tandem affinity purification‐mass spectrometry (TAP‐MS) to identify host factors that interact with σ3. We identified DHX9, a DEAD‐box/DEAH‐box helicase. DHX9 is expressed in both the cytoplasm and the nucleus, and it regulates multiple layers of the host's innate immune response. Thus, not surprisingly, several viruses hijack DHX9 to evade these responses [25, 26]. In some, but not all, cells, DHX9 serves as a cytoplasmic sensor of viral dsRNA or dsDNA, activating IFN expression [27]. However, in most cells, DHX9 is retained within the nucleus [28, 29]. Nuclear DHX9 is recruited to the NF‐κB promoter through an interaction with the p65 subunit of NF‐κB, where it acts to recruit RNA polymerase II to the promoter, thereby enhancing transcription [30]. Recent studies using myeloid‐ and hepatocyte‐specific DHX9 knockout mice have confirmed that DHX9 acts in the nucleus as a transcriptional coactivator to regulate ISG expression and counteract RNA viruses [28]. Regarding REOV infection, previous studies have shown that DHX9 pairs with MAVS to sense REOV dsRNA in myeloid dendritic cells [27]. However, the functional significance of nuclear DHX9 for REOV infection is unknown.

Here we identified DHX9 as a host restriction factor for REOV that facilitates NF‐κB activation, a function dependent on its helicase activity. The REOV σ3 protein functions as a vTR that suppresses NF‐κB activation through its interaction with DHX9. σ3 impairs the initial recruitment of Pol II by disrupting the interaction between DHX9 and Pol II. In addition, σ3 upregulates the R‐loop levels by inhibiting the helicase activity of DHX9, thereby affecting Pol II pause‐release and ultimately suppressing NF‐κB gene expression.

## 2 Results

### 2.1 Host DHX9 Interacts with REOV σ3

**FIGURE 1:**
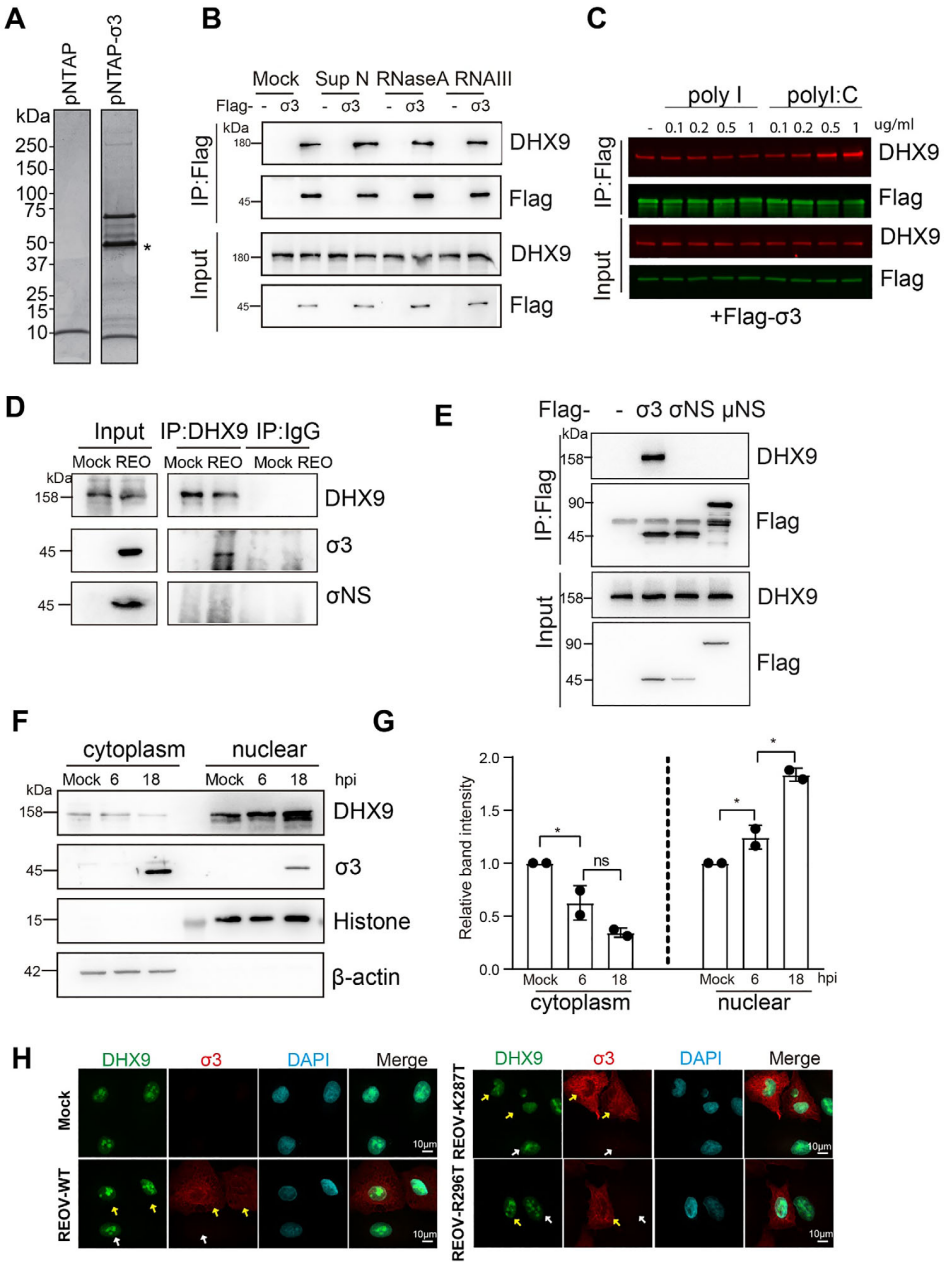
DHX9 is a host factor that interacts with σ3. (A) Purified proteins visualized by Coomassie blue staining. In HEK293T cells, Protein complexes associated with pNTAP‐σ3 were isolated by tandem affinity purification, separated by SDS‐PAGE, and visualized by Coomassie blue staining. Molecular weight marker is indicated on the left. The pNTAP empty vector served as a negative control. * indicates the bait protein σ3; (B) σ3 interacts with DHX9 in an DNA/RNA‐independent manner. HEK293T cells were transfected with either empty vector or pcDNA3.1‐Flag‐σ3. After 48 h, cells were harvested. Cell lysates were treated with or without RNase A (100 ng mL^−1^), Super Nuclease (25 U mL^−1^), or RNase III (10 U mL^−1^) at 37 °C for 20 min and then subjected for co‐IP; (C) The interaction between σ3 and DHX9 increases when cell lysates were treated with increasing amounts of poly I:C. Cell lysates from HEK293T cells were collected at 48 h post‐transfection and were then incubated with either poly I or poly I:C at the indicated concentrations on ice for 30 mins. After incubation, lysates were subjected for co‐IP using anti‐Flag agarose beads. (D) DHX9 interacts with σ3, but not σNS during REOV infection. HEK293T Cells were infected with REOV at a MOI of 10. At 18 hpi, cells were collected and lysed in the presence of Super Nuclease as described above. Co‐IP was then performed using anti‐DHX9 antibody. (E) σ3, but not µNS or σNS, specifically interacts with endogenous DHX9. (F) Subcellular localization of DHX9 or σ3 during the course of REOV infection. A549 cells were collected at 6 and 18 hpi, and then lysed for subcellular fractionation. Localization of indicated proteins in cytoplasm and nuclear fraction were assessed by western blotting. Histon H3 and Actin were used as marker for nuclear and cytoplasm fraction, respectively. (G) Relative band intensity was quantified for panel (F). Band intensities were all normalized to Mock sample. Data shown represent the mean ± s.d. of two independent experiments. Multiple non‐paired *t* test was used to analyze differences (ns = no significant, *p< 0.05). (H) DHX9 stays inside the nucleus in REOV infected cells. A549 cells were infected with REOV. At 18 h post infection, cells were fixed and co‐immunostained with rabbit anti‐DHX9 (in green) and mouse anti‐σ3 (in red). Yellow arrow indicates infected cells; white arrow indicates uninfected cells.

To explore the molecular mechanism by which σ3 regulates host transcription, we performed Tandem affinity purification followed by mass spectrometry (TAP‐MS) to identify host proteins that interacted with σ3. Following two rounds of affinity purification, multiple host proteins were found to co‐immunoprecipitate (co‐IP) with σ3 (Figure 1A and Data S1). Of these proteins, there are many components of the host chaperonin T‐complex protein‐1 (TCP‐1) ring complex that have been shown previously to interact with σ3 [31]. After excluding cytoskeletal proteins and chaperonins, among the top hits, we noted DHX9 as a protein that has previously been shown to be a sensor of REOV dsRNA during infection and to activate NF‐κB and IRF3 [27]. DHX9 is a member of the DEAD‐box/DEAH‐box helicase family and is involved in multiple cellular processes including transcriptional regulation. To investigate whether σ3 might regulate host transcription by interacting with DHX9, we first confirmed their interaction by coimmunoprecipitation (Co‐IP). Given that both DHX9 and σ3 are RNA‐binding proteins [32], we, therefore, tested their interaction under different enzymatic treatments (Super Nuclease S for digesting all nucleic acids; RNase A for digesting single‐stranded RNA; RNase III for digesting double‐stranded RNA). All nuclease treatments of the cell lysate did not disrupt the σ3‐DHX9 interaction, indicating a direct protein‐protein interaction (Figure 1B). We also tested whether dsRNA influenced the interaction between σ3 and DHX9. We found that addition of increasing amounts of poly I:C, a dsRNA analog, to the cell lysate enhanced the interaction between σ3 and DHX9 (Figure 1C). To assess if DHX9 interacted with σ3 during virus infection, we infected cells with REOV at a MOI of 10, and immunoprecipitated endogenous DHX9. σ3, but not another REOV protein σNS, co‐immunoprecipitated with DHX9 (Figure 1D). We further confirmed the specificity by individually expressing σ3, or σNS, or µNS. Only σ3, but not the two other REOV proteins, interacted with DHX9 (Figure 1E).

To further examine DHX9 dynamics during REOV infection, we analyzed its subcellular localization in cells infected with REOV. Similar to the viral protein σ3, DHX9 exhibited both nuclear and cytoplasmic distribution (Figure 1F), with significantly lower intensity in the cytoplasm. Notably, DHX9 levels gradually increased in the nuclear fraction, while decreasing in the cytoplasmic fraction throughout infection, indicating a translocation of DHX9 from the cytoplasm to the nucleus (Figure 1F,G). When we co‐stained the cells with antibodies against DHX9 and σ3 upon infection with either wild‐type REOV or two σ3 mutant viruses that were identified in our previous study [24], DHX9 did not localize to the cytoplasm, or form cytoplasmic puncta, which contrasts with the previous reports that DHX9 pairs with MAVS in the cytoplasm of REOV‐infected myeloid dendritic cells to sense dsRNA [27] (Figure 1H). Collectively, these data highlight that DHX9 is a host factor that interacts with σ3, suggesting a potential role for DHX9 within the nucleus during infection.

### 2.2 σ3 WT, but Not the K287T Mutant, Interacts with the Helicase Domain of DHX9

**FIGURE 2:**
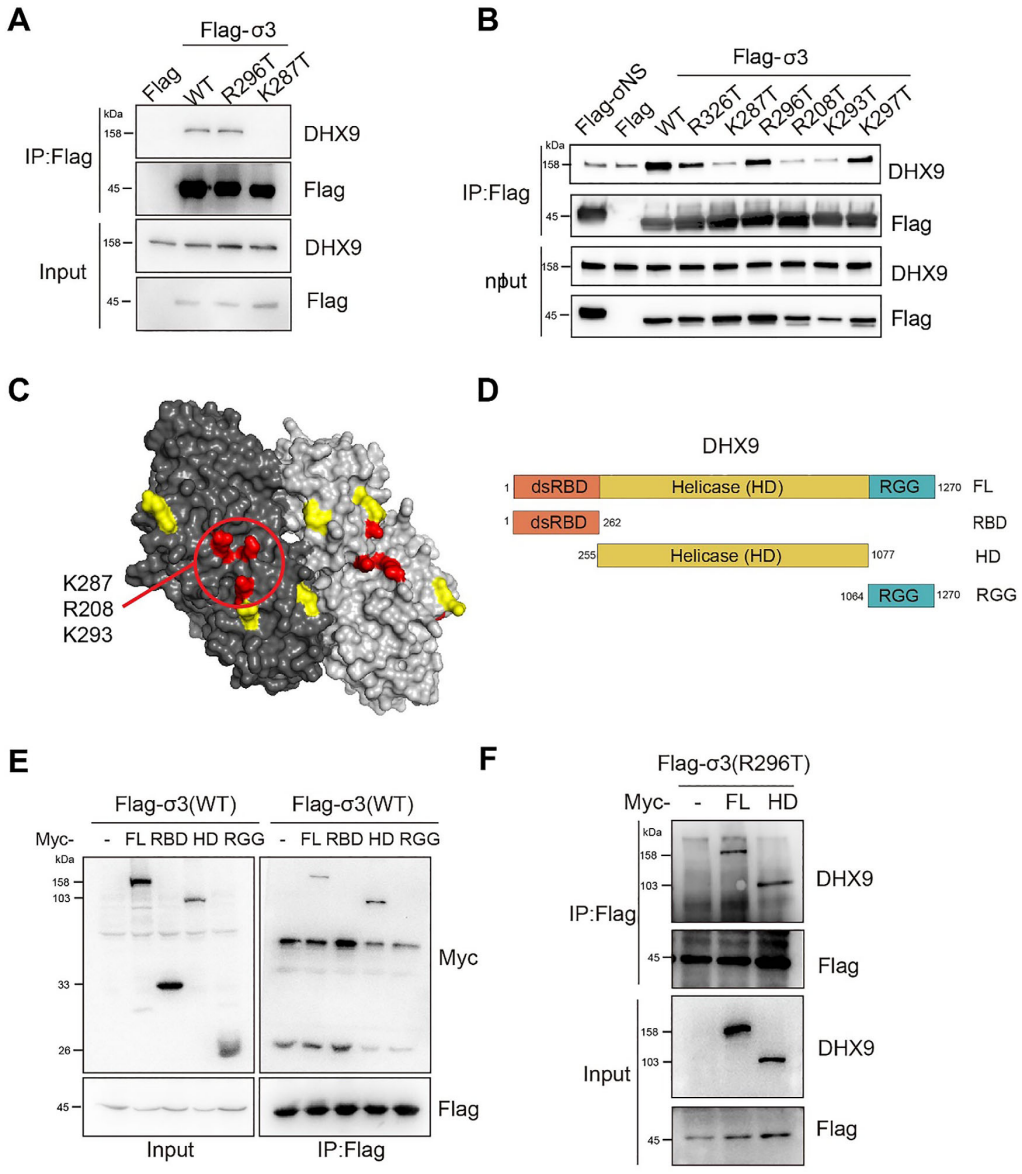
Characterization of the interaction between σ3 and DHX9. (A,B) σ3 mutants K287T, R208T, and K293T did not interact with DHX9. (C) Space‐filling surface view of the σ3 dimer structure (PDB ID: 1FN9) showing the mutated residues. Mutated residues in Red indicates disrupted the σ3‐DHX9 interaction. Residues colored yellow are dsRNA‐binding defective but are capable of binding DHX9. (D) Schematic representations of full‐length and the indicated truncations of DHX9. HD: Helicase domain; RBD: dsRNA binding domain; RGG: C‐terminal RGG‐containing domain. Numbers denote amino acid residues. (E) The helicase domain of DHX9, but not the RBD or RGG domains, interacts with σ3. Myc‐tagged DHX9 (full length or truncated) was transfected together with Flag tagged σ3 into HEK293 cells. Co‐IP was performed 48 h post‐transfection using anti‐Flag beads, followed by immunoblotting to detect the indicated proteins. (F) Full length DHX9 or its helicase domain (HD) interact with the σ3 R296T mutant.

We previously identified mutants of σ3 that do not bind dsRNA [24]. Because dsRNA enhanced the interaction of DHX9 with σ3, we tested whether two σ3 dsRNA‐binding mutants, K287T and R296T, could interact with DHX9. Recombinant viruses with the R296T mutation in σ3 have a WT phenotype in vitro and in vivo. In contrast, the K287T mutant has defects in viral replication, stress granule formation, PKR activation, and the capacity to induce myocarditis in a mouse model [24]. We found that like WT σ3, the R296T mutant retained the capacity to interact with DHX9, indicating that dsRNA‐binding by σ3 was not required for the interaction (Figure 2A). However, interestingly, we found that the K287T mutant did not interact with DHX9 (Figure 2A). To investigate further, we assessed other dsRNA binding mutants that we identified in our previous study. Although all the σ3 mutants exhibited subcellular localization pattern similar to the wild‐type protein (Figure S1), three dsRNA‐binding mutants, K287T, R208T and K293T were unable to interact with DHX9 (Figure 2B). These three residues are located close to each other on the surface of the σ3 structure, forming a possible DHX9 binding patch on σ3 (Figure 2C).

To map the domain(s) of DHX9 that interact with σ3, we divided DHX9 into three functional domains: the dsRNA binding domain (RBD; amino acids 1–262), the helicase domain (HD; amino acids 255–1077), and the RGG domain (rich in arginine repeat sequence; amino acids 1064–1270) as described previously [33] (Figure 2D). We found that only the helicase domain of DHX9 interacted with σ3 (Figure 2E). To further confirm that dsRNA was not required for the interaction of DHX9 and σ3, we tested whether the σ3 R296T mutant interacted with the helicase domain of DHX9. We found that the interaction was comparable to that of wild‐type DHX9, suggesting that the helicase domain is sufficient for the σ3‐DHX9 interaction, and that although dsRNA enhances the interaction, it is not required for it (Figure 2E,F).

### 2.3 DHX9 acts as a Host Restriction Factor of REOV through Its Helicase Activity

**FIGURE 3:**
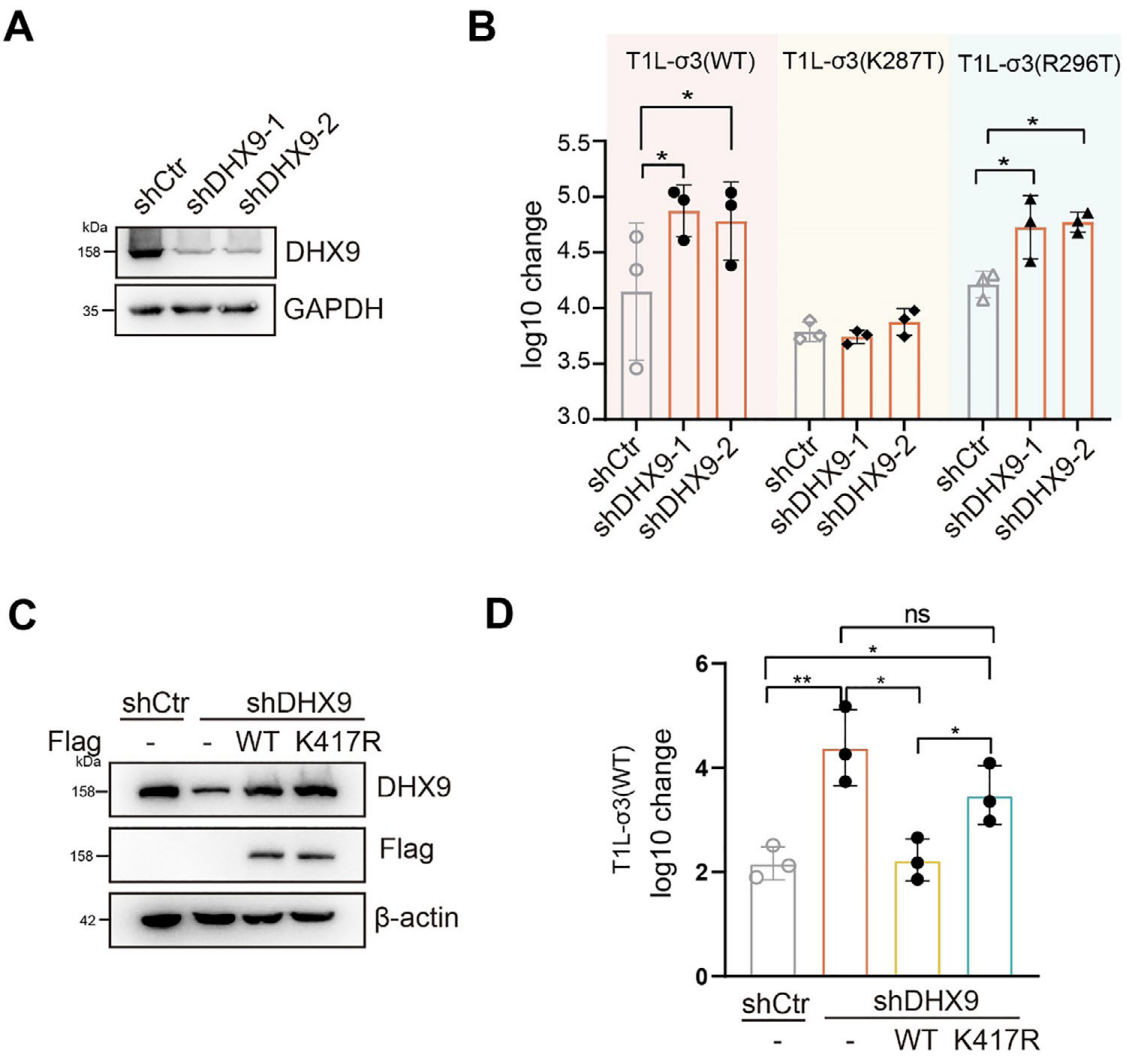
DHX9 acts as a host restriction factor for REOV through its helicase activity. (A,B) T1L‐WT and T1L‐R296T viruses replicated more efficiently in DHX9‐deficient A549 cells, whereas T1L‐K287T did not. A549 cells were first infected with lentivirus encoding shRNA against DHX9. After 48 h, cells then infected with the indicated REOV at MOI 10. Viral growth was shown as changes in viral titer from 0 to 24 h pi. The efficiency of DHX9 knockdown was verified by western blotting in (A). Data shown represent the mean ± s.d. of three independent experiments. (C,D) Viral growth of T1L‐WT was assessed after reconstitution of either WT DHX9 or its helicase dead mutant K417R in A549 cells deficient in DHX9. The expression levels of DHX9 were examined by western blotting in (C). Viral growth was shown as changes in viral titer from 0 to 24 h pi. Data shown represent the mean ± s.d. of three independent experiments. Multiple non‐paired *t* test was used to analyze differences (ns = no significant, *p< 0.05, **p< 0.01).

To assess whether DHX9 played a role in suppressing viral replication, we knocked down DHX9 in A549 cells using shRNA (Figure 3A). Upon DHX9 knockdown, we observed increased replication of the WT and R296T viruses, but not the K287T virus, indicating that endogenous DHX9 restricts REOV replication (Figure 3B). Similarly, the K293T virus, which also fails to interact with DHX9 (Figure 2B), exhibited viral replication comparable to the K287T virus in both mock and DHX9‐depleted cells (Figure S2), suggesting that the residues at positions 293 and 287 may be functionally similar.

As σ3 interacted with the helicase domain of DHX9, we asked whether the helicase activity of DHX9 was required to limit REOV replication. To address this question, we reconstituted the DHX9 knock‐down cells with either wild‐type DHX9 or a helicase‐dead mutant, K417R (Figure 3C). We found that wild‐type DHX9, but not the helicase‐dead mutant, reduced the upregulated virus titer observed following DHX9 depletion. A significant difference in viral replication was observed between WT and K417R reconstituted cells, indicating that the helicase activity of DHX9 is required for its anti‐viral function (Figure 3D). To further validate this finding, we treated cells with a specific DHX9 inhibitor, ATX968 [34], and found that DHX9 suppression enhanced viral replication (Figure S3), further supporting that DHX9 restricted REOV replication via its helicase activity.

### 2.4 σ3 Inhibits DHX9 Helicase Activity In Vitro

**FIGURE 4:**
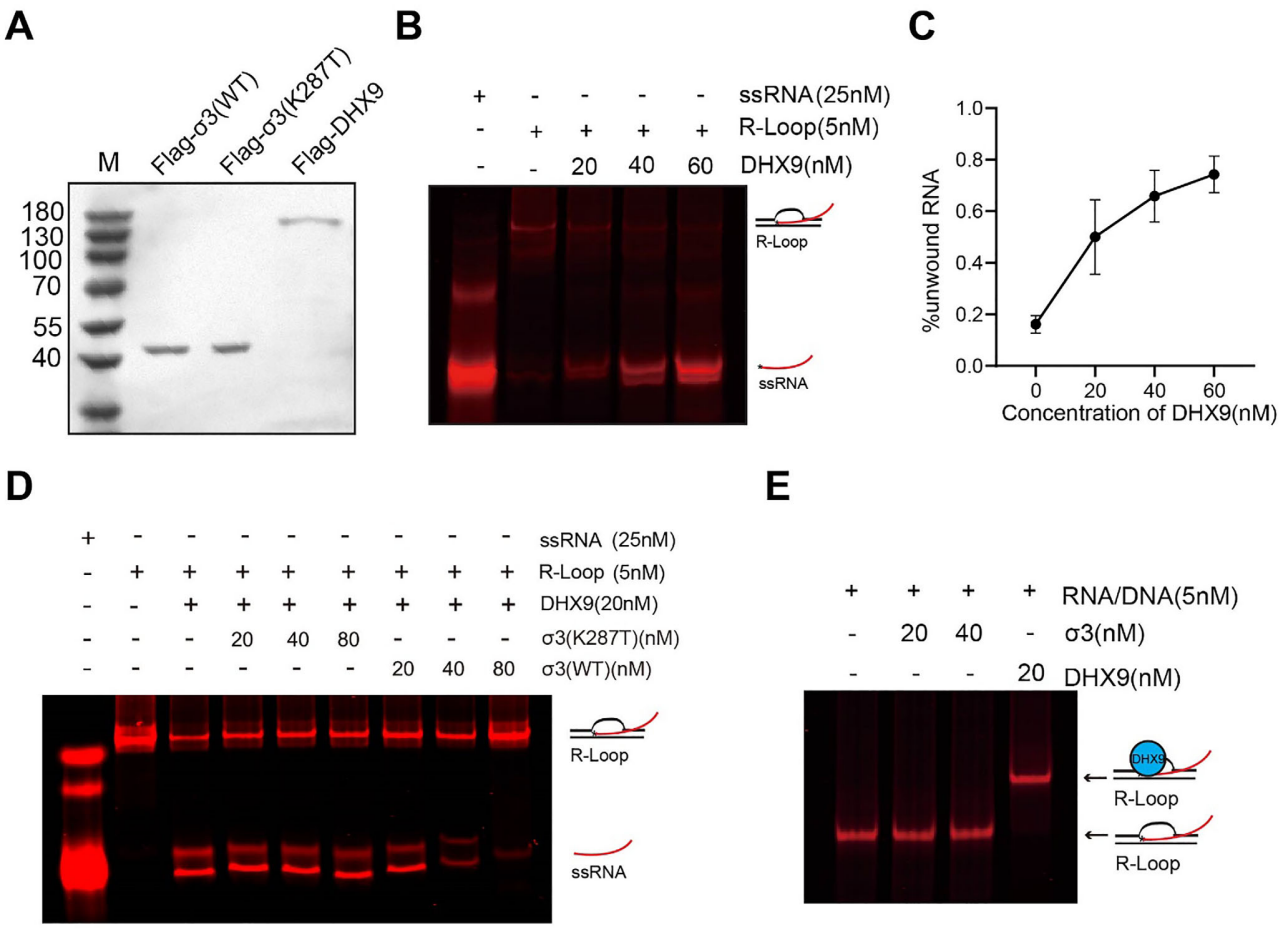
σ3 inhibits DHX9 helicase activity in vitro. (A) Coomassie Blue staining of recombinant σ3 (WT/K287T) and DHX9 purified from 293F cells. (B) DHX9 resolves R‐loops in vitro. The in vitro helicase assay on R‐loops was performed by incubating the 5’ Cy5.5‐labeled R‐loop substrates with increasing amounts of recombinant DHX9 at 37 °C for 10 min, and subsequently analyzed by native polyacrylamide gel electrophoresis and fluorescence imaging. (C) Quantification of DHX9's unwinding activity. Error bars indicate SD as derived from three independent experiments. (D) σ3 WT, but not K287T, inhibits the helicase activity of DHX9. DHX9 was incubated with increasing amounts of either σ3 WT or K287T at room temperature for 30 min, and then subjected for helicase assay by adding R‐loop substrates and ATP. (E) DHX9, but not σ3 binds R‐loops. Indicated amounts of DHX9 or σ3 were incubated with R‐loop substrates at 37 °C for 15 min. The reaction was then terminated and loaded onto native PAGE gels to check the band shift. All images were collected using Licor Odessey imaging system.

To investigate how DHX9 exerts its anti‐viral function, we revisited the interaction between σ3 and DHX9. Given that the helicase activity of DHX9 was required for its anti‐viral function, and that σ3 interacted with the helicase domain, we hypothesized that σ3 inhibited DHX9 helicase activity. To address this hypothesis, we expressed and purified DHX9 and σ3 proteins (both WT and the K287T mutant) (Figure 4A) and tested if σ3 inhibited DHX9 helicase activity in vitro. Previous studies have demonstrated that R‐loops with 3' overhangs are particularly sensitive to DHX9 [35], therefore, we generated such structures by annealing two single‐stranded DNA oligos and one single‐stranded RNA oligo, and verified the presence of the hybrid structure with a monoclonal antibody (S9.6) that recognizes R‐loops [36, 37]. As the concentration of R‐loop substrates increased, the band intensity of S9.6 gradually increased (Figure S4A). When increasing concentrations of DHX9 were added to the pre‐formed R‐loops, the R‐loops were unwound, as evidenced by the appearance of resolved ssRNA (Figure 4B,C) and decreased S9.6 slot blot signal (Figure S4B). We also assessed the kinetics of DHX9 helicase activity using increasing concentrations of R‐loop substrates. We calculated a *K*m value of 15.1 nM (Figure S4C,D). Interestingly, when increasing concentrations of σ3 were added to the reaction, the S9.6 signal gradually recovered (Figure S4E), and the amount of ssRNA resolved by DHX9 gradually decreased (Figure 4D), indicating that σ3 inhibits the helicase activity of DHX9 in a dose‐dependent manner. In contrast, the addition of the σ3 K287T mutant, which did not bind DHX9, did not affect the capacity of DHX9 to resolve R‐loops (Figure 4D). To rule out the possibility that σ3 inhibits DHX9 from unwinding R‐loops by competing for R‐loop substrates, we tested whether σ3 could bind R‐loops independently of DHX9. At 20 nM, DHX9 efficiently binds R‐loops, as demonstrated by the shift of R‐loops, whereas σ3 did not cause this shift under any of the concentrations tested (Figure 4E). Collectively, our findings show that σ3 interacts with DHX9 and inhibits its helicase activities in vitro.

### 2.5 σ3 Inhibits NF‐κB‐Dependent Gene Expression Through Its Interaction With DHX9

σ3 has been implicated in transcriptionally regulating NF‐κB‐dependent gene expression [21]. However, the molecular mechanism remains unknown. Given the critical role of DHX9 in mediating NF‐κB‐dependent gene transcription, we hypothesized that σ3 might inhibit NF‐κB‐dependent gene transcription by interacting with DHX9.

**FIGURE 5:**
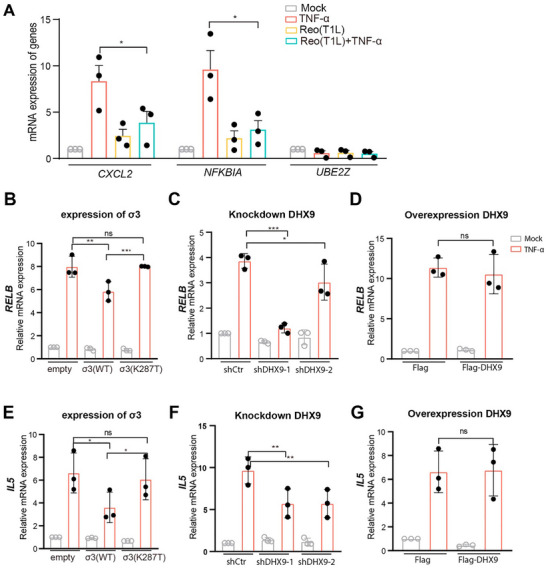
σ3 inhibits TNF‐α‐stimulated NF‐κB gene expression through its interaction with DHX9. (A) REOV inhibits TNF‐α‐stimulated NF‐κB gene expression. HEK293T cells were infected with T1L at MOI 10. At 18 h pi, cells were treated with or without TNF‐α at 10 ng mL^−1^ for 1 h. RNA was extracted from cells, and the levels of *CXCL2*, *NFKBIA* and *UBE2Z* mRNA relative to *GAPDH* were measured by RT‐qPCR and comparative ΔΔC_T_ analysis. (B‐G) The mRNA levels of *RELB* and *IL5* relative to *GAPDH* were measured by RT‐qPCR after σ3 expression (B and E), or DHX9 knockdown (C and F), or DHX9 overexpression (D and G) in HEK293T. Data are presented as the mean ± s.d. of three independent experiments. Non paired *t* test was used to analyze the differences (ns = no significant, *p< 0.05, **p< 0.01, ***p<0.001).

To confirm that REOV infection reduced NF‐κB‐dependent gene expression, we examined two genes upregulated by NF‐κB signaling: *NFKBIA* which encodes the I‐kappaB‐alpha and *CXCL2* which encodes the cytokine CXCL2. Upon treatment of cells with TNF‐α, an NF‐κB agonist, we found that REOV infection significantly reduced TNF‐α‐induced expression of *NFKBIA* and *CXCL2*, whereas the level of a control gene, *UBE2Z*, was unchanged (Figure 5A).

To determine if the interaction between σ3 and DHX9 was required for the changes in gene expression, we compared the effect of WT σ3 or the K287T mutant on NF‐κB‐dependent gene expression. We assessed the expression of the NF‐κB‐dependent genes *RELB and IL5* following TNF‐α treatment of cells expressing σ3. We found that unlike WT σ3, the K287T mutant failed to reduce these two genes, indicating that the interaction of σ3 with DHX9 is required for σ3 to regulate these NF‐κB‐dependent genes (Figure 5B,E). We found that expression of both genes was significantly decreased when DHX9 was depleted using shRNA (Figure 5C,F) but remained unchanged when DHX9 was overexpressed (Figure 5D,G). In summary, these results indicate that the interaction between σ3 and DHX9 is essential for σ3 to suppress NF‐κB‐dependent gene expression.

### 2.6 Wild‐Type σ3, but Not K287T, Impairs both the DHX9‐Dependent Recruitment and the Pause‐Release of RNA Polymerase II (Pol II)

**FIGURE 6:**
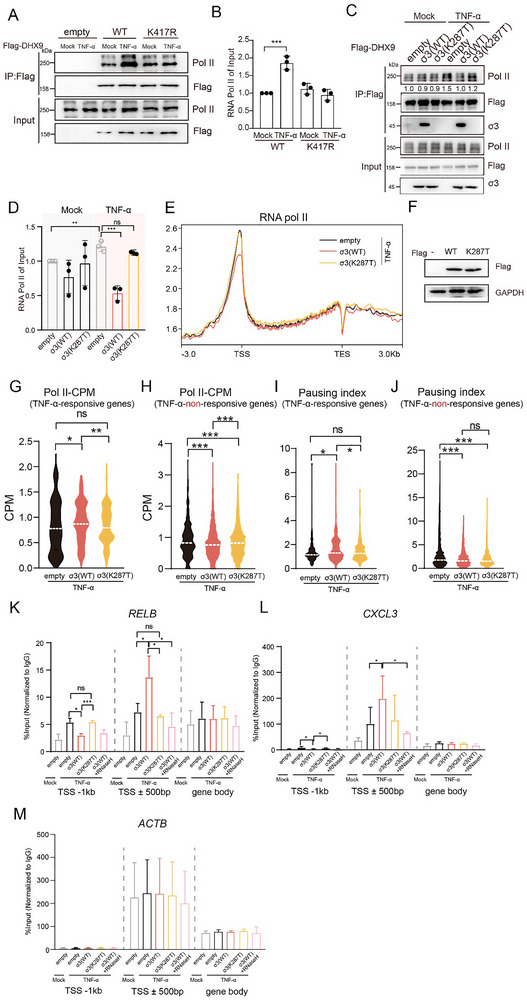
Wild‐type σ3, but not K287T, impairs both the DHX9‐dependent recruitment and the pause‐release of RNA polymerase II (Pol II). (A) DHX9 helicase activity is required for the increased interaction between DHX9 and RNA Pol II upon TNF‐α treatment. Co‐IP experiment was performed in HEK293T between exogenously expressed Flag‐tagged DHX9 (either WT or K417R) and endogenous RNA pol II following TNF‐α treatment. The co‐IP efficiency was quantified by comparing the band intensity in the IP lane to the band in the input lane. All samples were normalized to mock (set at 1). Quantification of three independent experiments were shown in (B); (C) expression of σ3 WT, but not K287T, significantly reduced the interaction between DHX9 and RNA pol II upon TNF‐α treatment. The co‐IP efficiency was quantified by comparing the band intensity in the IP lane to the band in the input lane. All samples were normalized to mock (set at 1). Quantification of three independent experiments were shown in (D). (E) Average Pol II CUT & Tag signal profile on genomic loci (defined as 3 kb upstream of annotated TSS to 3 kb downstream of annotated TES) in HEK293T cells expressing σ3 or K287T upon TNF‐α treatment from two biological replicates. (F)Western blotting shows the equal expression levels of σ3(WT) and σ3(K287T). (G,H) Comparison of Counts per Million (CPM) of Pol II at promoter proximal region (TSS ± 500 bp) for (G) TNF‐α‐responsive genes (*n* = 110) or (H) TNF‐α‐non‐responsive genes (*n* = 896). Paired *t*‐tests were used to evaluate the signal differences. (I,J) Pausing index of (I) TNF‐α‐responsive genes or (J) TNF‐α‐non‐responsive genes. (K‐M) ChIP‐qPCR analysis of Pol II enrichment at specific genomic regions (−1 kb from the TSS, ± 500 bp around TSS, and within the gene body) of *RELB*, *CXCL3*, and *ACTB* in HEK293T cells expressing σ3 upon TNF‐α treatment. The condition labeled “σ3+RNase H” represents cells coexpressing σ3 and RNase H prior to TNF‐α treatment. Pol II enrichment was determined using the percent input method. Data are presented as the mean ± s.d. of three independent experiments. Paired *t* test was used to analyze the differences (ns = no significant, *p< 0.05, **p< 0.01, ***p<0.001).

DHX9 mediates the recruitment of RNA polymerase II to the NF‐κB promoter region [30]. Whether the helicase activity of DHX9 is required for this interaction remains unclear [30, 38]. To investigate how the interaction of σ3 with DHX9 modifies transcriptional regulation of NF‐κB‐dependent gene expression, we first examined the interaction between FLAG‐tagged DHX9, or its helicase‐dead mutant K417R, with endogenous RNA pol II. Under resting conditions, FLAG‐tagged DHX9 and the K417R showed similarly levels of interaction with Pol II. However, after TNF‐α treatment, the interaction between WT FLAG‐tagged DHX9 and Pol II increased, whereas the interaction of the helicase‐dead mutant K417R remained at the baseline level (Figure 6A,B). These findings suggest that following TNF‐α treatment, the helicase activity of DHX9 is required to enhance its interaction with Pol II. We next asked if σ3 expression would disrupt the TNF‐α‐induced interaction of DHX9 with Pol II. We expressed either σ3 or K287T and evaluated the DHX9‐Pol II interaction by co‐IP. We found that expression of WT σ3, but not the K287T mutant, decreased the interaction between DHX9 and Pol II (Figure 6C,D), indicating that interaction of σ3 with DHX9 decreases the TNF‐α‐induced interaction of DHX9 with Pol II.

The co‐IP experiments described above (Figure 6A–D) did not distinguish between free Pol II in the nucleoplasm and chromatin‐bound Pol II that is actively engaged in transcription. To better understand how σ3 affects Pol II occupancy on chromatin at the single gene level, we conducted CUT & Tag experiments (Cleavage Under Targets and Tagmentation). We transfected cells with either empty vector or plasmids expressing either σ3 WT or K287T and then checked the Pol II occupancy after TNF‐α treatment. Consistent with previous findings [39], we found Pol II enriched predominantly in regions near transcriptional start sites (TSS) and transcriptional termination sites (TES) (Figure 6E). Upon expression of WT σ3, but not the K287T mutant, the global occupancy of Pol II on chromatin was notably decreased (Figure 6E,F; Figure S5A,B). Interestingly, we identified a subset of genes that were silenced by σ3 expression (Data S2). Notably, we found that several of these silenced genes are involved in antiviral immune responses, such as APOBEC3G, ZNF841, LILRA1, TMEM45B, CR1, etc. A complete list of these genes is provided in the Data S2, which we believe represent interesting candidates for future investigation.

To determine the effect of σ3 on NF‐κB‐dependent genes, considering that NF‐κB‐dependent gene expression is highly stimulus‐ and cell type‐specific [40, 41, 42, 43], we performed RNA‐seq to identify genes that are responsive to TNF‐α under our experimental conditions. Eventually, we identified 110 TNF‐α‐responsive genes and 896 TNF‐α‐non‐responsive genes (Data S3). Surprisingly, we observed that upon expression of σ3, but not K287T mutant, Pol II occupancy in the promoter proximal region (‐500 bp to +500 bp) was upregulated in TNF‐α–responsive genes, but downregulated in TNF‐α–non‐responsive genes (Figure 6G,H; Figure S5C,D).

Pausing of Pol II represents a widespread regulatory mechanism in higher eukaryotes, serving as a critical regulatory step beyond initial Pol II recruitment. Previous study has reported that TNF‐α‐stimulated NF‐κB signaling leads to release of paused Pol II from the promoter‐proximal pause site, thereby promoting efficient transcription elongation [44]. To further investigate, we calculated the pausing index (PI) as described previously [45, 46]. Interestingly, we observed that PI increased in TNF‐α‐responsive genes but decreased in TNF‐α‐non‐responsive genes upon σ3 expression (Figure 6I,J). To further validate this finding, we performed ChIP‐qPCR using primers designed to amplify three different regions (TSS‐1 kb, TSS±500 bp, and gene body) of *CXCL3*, *RELB*, and *ACTB*. Interestingly, the expression of σ3, but not K287T, significantly decreased the Pol II enrichment at TSS‐1 kb region of *RELB* and *CXCL3*. However, for the promoter proximal region (TSS±500 bp), the expression of σ3, but not K287T, significantly upregulated the Pol II enrichment onto *RELB* and *CXCL3*, but not *ACTB* (Figure 6K–M).

Collectively, our findings indicate that σ3 expression impairs the pause‐release of Pol II at the promoter‐proximal sites, thereby repressing the expression of TNF‐α‐responsive genes. Conversely, for TNF‐α‐non‐responsive genes, σ3 appears to exert a general repressive effect on transcription by decreasing the Pol II at the promoters through a mechanism that remains to be elucidated.

### 2.7 REOV Infection or σ3 Expression Leads to Increased Levels of Cellular R‐Loops

**FIGURE 7:**
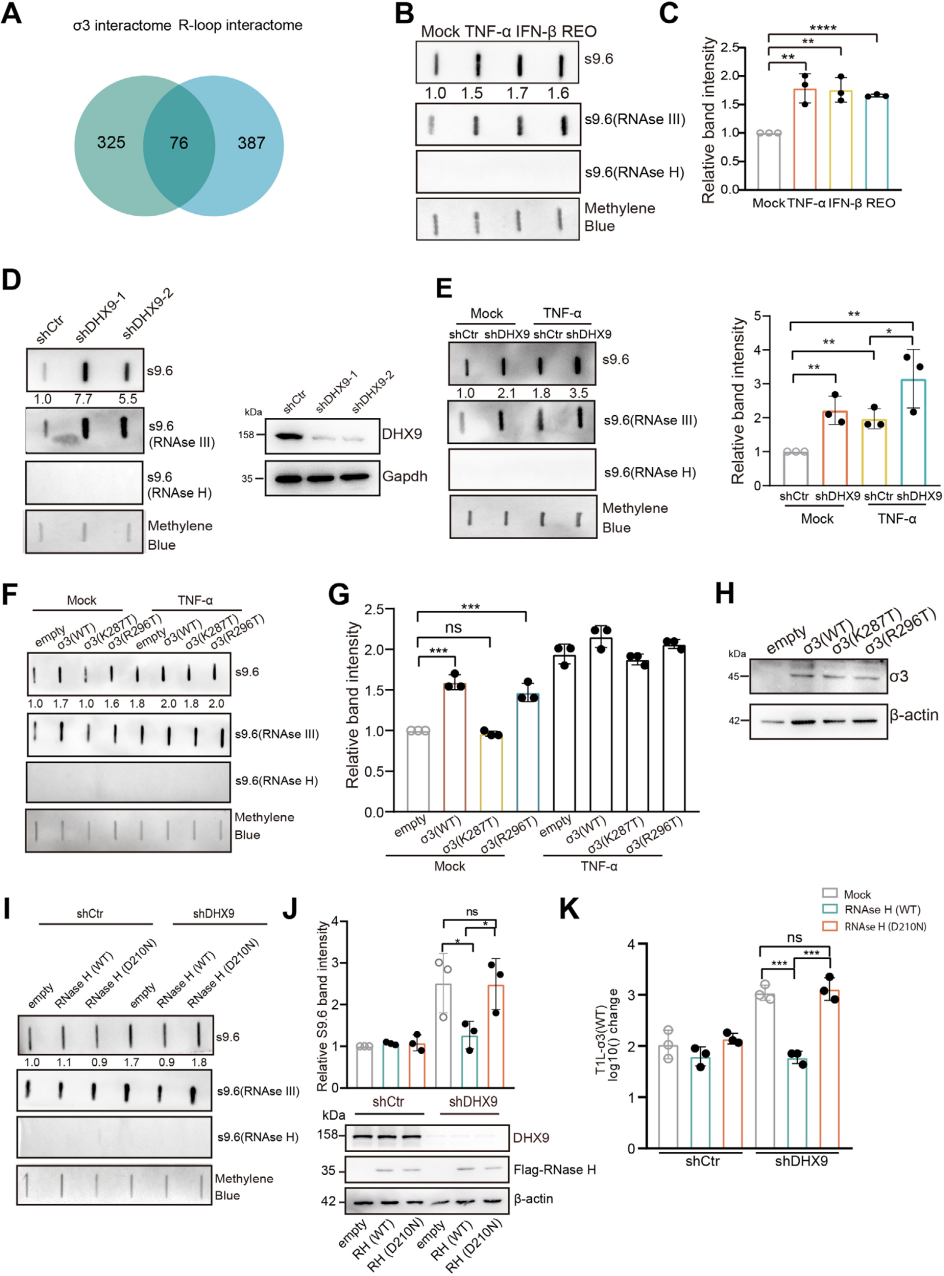
REOV infection, or σ3 expression, leads to an upregulation of cellular R‐loops, potentially contributing to viral replication. (A) Venn diagram showing the overlap of σ3 interactome (green) with known interactors of R‐loops (blue). (B) Cellular R‐loop levels were upregulated upon treatment of TNF‐α, IFN‐β, or REOV infection. A549 cells were treated with IFN‐β (10 ng mL^−1^), TNF‐α (10 ng mL^−1^) for 3 h or infected with REOV at MOI 50 for 6 h. Genomic DNA were then extracted, and R‐loop levels were detected using slot blot assay with the S9.6 antibody. (C) Quantification of three independent experiments as shown in (B). Relative S9.6 band intensity was first normalized to methylene blue staining, and then R‐loop level in mock was set as 1. (D,E) Knocking down DHX9 led to an upregulation in cellular R‐loop levels in Mock (D) or cells treated with TNF‐α (E). A549 cells were infected with lentivirus encoding shRNA against DHX9. At 48 h pi, cells were either mock‐treated or treated with TNF‐α. Genomic DNA isolated from nuclear extracts were analyzed for R‐loops using slot blot assay with the S9.6 antibody. (F) σ3 expression led to an upregulation in cellular R‐loop levels. Band intensity quantification of three independent experiments was shown in (G). (H) Western blotting showed the equal expression levels of σ3. (I,J) Cellular levels of R‐loops and (K) Viral growth of T1L‐WT, were assessed after overexpression of either WT RNase H or its enzymatically dead mutant D210N in A549 cells deficient in DHX9. Viral growth was shown as changes in viral titer from 0 to 24 h pi. Data shown represent the mean ± s.d. of three independent experiments. One‐way ANOVA followed by Tukey post hoc analysis was used for statistical analysis (ns = no significant, *p< 0.05, **p< 0.01, ***p<0.001, ****p<0.0001).

Increasing evidence highlights R‐loops as essential players in gene regulation [12, 14, 15]. When we compared the σ3 interactome (consisting of 401 candidate proteins) with the previously published R‐loop interactome (composed of 463 candidate proteins) [47], we identified an overlap of 76 proteins (Figure 7A and Data S1), including PRKDC, DHX9, ILF2, ILF3, NPM1, NCL, HNRNPL, etc. This observation led us further to investigate the impact of R‐loops on viral replication. To explore the relationship between R‐loops and innate immune responses, we first assessed the cellular levels of R‐loops upon REOV infection or stimulation with TNF‐α or IFN‐β. To exclude any potential nonspecific recognition of dsRNAs by S9.6, as reported previously in immunofluorescence staining assays, we treated the extracted genomic DNAs with either RNase III to remove dsRNA, or RNase H to digest R‐loops as a negative control, before proceeding with S9.6 slot blotting. We found that REOV infection, as well as treatment with TNF‐α or IFN‐β, all led to increased cellular levels of R‐loops (Figure 7B,C). We also utilized a previously established immunofluorescence assay employing a GFP‐tagged catalytically inactive RNase H mutant (dRNase H:D210N). This mutant recognizes R‐loops but lacks enzymatic activity. Imaging with GFP‐dRNase H revealed similar results consistent with the slot blot assay that TNF‐α treatment or REVO infection significantly increased cellular R‐loop levels (Figure S6). Interestingly, we noted that a subset of R‐loop hotspots overlaps across three conditions (TNF‐α, or IFN‐β, and REOV infection) (Figure S7), which we are going to investigate in the future.

To assess the role of DHX9 in regulating cellular R‐loop levels, we evaluated the effect of depleting DHX9 in mock or TNF‐α‐treated cells. DHX9 depletion resulted in a significant upregulation of R‐loops, supporting the role of DHX9 in resolving R‐loop structures (Figure 7D). Interestingly, knockdown of DHX9 resulted in an even further upregulation of cellular R‐loop levels in TNF‐α‐treated cells (Figure 7E).

Given these observations, we asked what effect σ3 would have on R‐loop levels. We found that WT σ3 and the R296T mutant but not the DHX9‐binding defective mutant K287T, led to increased cellular R‐loop levels (Figure 7F–H), consistent with the observed inhibition of DHX9 helicase activity by σ3 in vitro (Figure 4D). However, the effect of σ3 was eliminated in cells treated with TNF‐α (Figure 7F–H). Taken together, these findings suggest that REOV infection, or the expression of σ3, increases cellular R‐loop levels, and this is due in part to the capacity of σ3 to inhibit DHX9 helicase activity.

To further explore the impact of cellular R‐loops on viral replication, we overexpressed RNase H, a host enzyme that specifically resolves R‐loop structures. In control cells, we did not observe a substantial change in R‐loop levels upon overexpression of RNase H (Figure 7I,J). Upon depletion of DHX9 using shRNAs, cellular R‐loop levels increased. In DHX9‐depleted cells, overexpression of RNase H, but not its enzymatically inactive mutant D210N, restored the elevated R‐loop level back to baseline (Figure 7I,J). To examine the role of R‐loops in virus replication, we found that REOV replicated more efficiently in cells deficient in DHX9. Notably, the replication‐promoting effect of DHX9 knockdown was reversed by the overexpression of RNase H. In contrast, overexpression of the RNase H D210N mutant did not affect viral replication in DHX9‐deficient cells (Figure 7K). These results suggest that elevated cellular levels of R‐loops facilitate virus replication.

### 2.8 σ3 Expression, Leads to the Upregulation of R‐Loops at the Promoter‐Proximal Region of NF‐κB‐Dependent Genes

**FIGURE 8:**
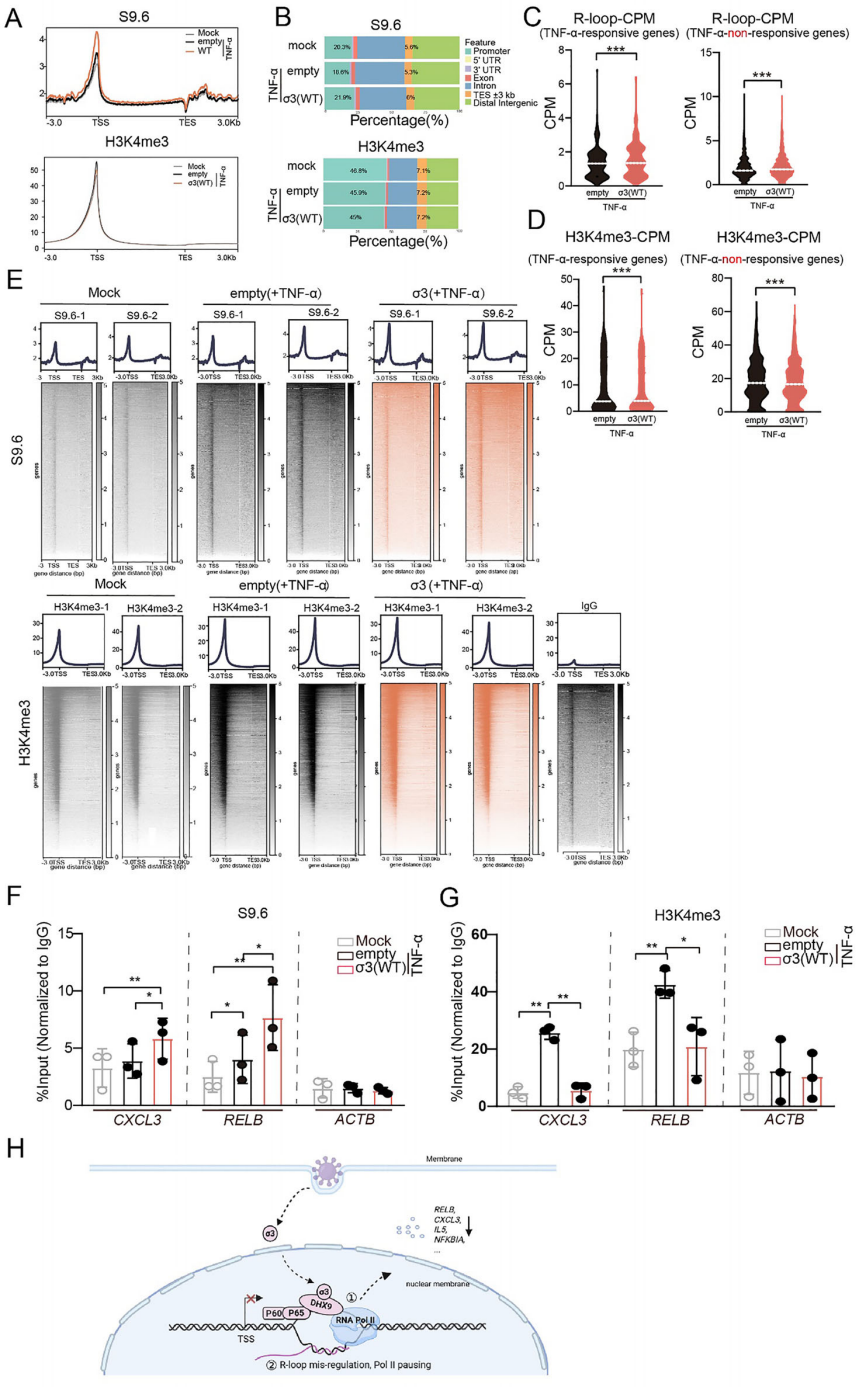
σ3 expression, leads to an upregulation of R‐loops at the promoter proximal region of TNF‐α‐responsive genes. (A) Average R‐loop (upper) and H3K4me3 (lower) CUT & Tag signal profile on genomic loci (defined as 3 kb upstream of annotated TSS to 3 kb downstream of annotated TES) in HEK293T cells expressing σ3 upon TNF‐α treatment. (B) Genome‐wide distribution of R‐loop (upper) and H3K4me3 (lower) peaks. (C and D) Comparison of abundance of R‐loop (C) and H3K4me3 (D) at promoter proximal region of TNF‐α‐responsive genes or TNF‐α‐non‐responsive genes. (E) Heatmaps show the read density that is ranked by decreasing occupancy of R‐loop (upper) and H3K4me3 (lower). (F,G) ChIP‐qPCR analysis of R‐loop (F) or H3K4me3 (G) enrichment at the promoter proximal region of *RELB*, *CXCL3*, and *ACTB* in HEK293T cells expressing σ3 upon TNF‐α treatment. Enrichment is calculated by percent input method. Data are presented as the mean ± s.d. of three independent experiments. Paired *t* test was used to analyze the differences (ns = no significant, *p< 0.05, **p< 0.01, *** *p*<0.001). (H) A working model of how σ3 inhibits NF‐κB gene expression (created using BioRender.com): ① σ3 competes with Pol II for interaction with DHX9, thereby decreasing the DHX9‐dependent recruitment of Pol II to NF‐κB‐dependent promoters. Meanwhile/subsequently, ② σ3 upregulates the R‐loop levels by inhibiting the helicase activity of DHX9, thereby affecting Pol II pause‐release and ultimately suppressing NF‐κB gene expression.

Although the above results indicated a potential correlation between host R‐loop levels and viral replication, it was unclear how increased R‐loop formation benefited viral replication. Given that σ3 transcriptionally regulates NF‐κB to antagonize the host innate immune response, we explored whether R‐loops might be involved in this process. To this end, we conducted CUT & Tag using the S9.6 antibody to profile genome‐wide R‐loops following TNF‐α treatment. As described previously [15], R‐loops are mainly enriched in gene promoter‐proximal regions and terminator regions. Notably, when σ3 was expressed, there was an increase in the abundance of R‐loops at promoter proximal regions (Figure 8A). When we analyzed the genome‐wide distribution of R‐loops, we observed a higher percentage of R‐loop occupancy in the promoter proximal region in cells expressing σ3 (21.9% vs. 18.6% in empty vector‐transfected cells) (Figure 8B). When we analyzed the R‐loops abundance in promoter proximal regions of TNF‐α‐responsive genes, with or without σ3 expression, we found that σ3 expression led to a small but significant increase in R‐loops at the promoter proximal regions in both TNF‐α‐responsive genes and TNF‐α‐non‐responsive genes, aligning with the observed upregulation of cellular R‐loop upon σ3 expression (Figure 8C,E).

Trimethylation of histone H3 lysine 4 (H3K4me3) is associated with transcriptional start sites and has been proposed to promote transcription. Therefore, we also assessed the status of H3K4me3 by CUT & Tag as an alternative means to evaluate transcription. As reported previously, H3K4me3 predominantly occurs at promoter regions [48]. Although we did not observe global changes in the abundance or the distribution of H3K4me3 across different gene regions, the abundance of H3K4me3 at TNF‐α‐responsive genes promoters was significantly reduced upon σ3 expression, which correlates with the transcriptional downregulation of these genes by σ3 (Figure 8A,B,D,E).

To further validate these findings, we performed ChIP‐qPCR to assess R‐loop and H3K4me3 enrichment at the promoters of *CXCL3*, *RELB*, and *ACTB*. σ3 expression significantly upregulated R‐loop enrichment at the *RELB* and *CXCL3* promoters, but not at the *ACTB* promoter. TNF‐α treatment significantly increased the H3K4me3 abundance at the *CXCL3* and *RELB* promoters, but not at *ACTB*. Furthermore, expression of σ3 significantly reduced the H3K4me3 enrichment at both the *CXCL3* and *RELB* promoters (Figure 8F,G).

Previous studies suggest that the excessive R‐loops at the promoter region might affect Pol II pause‐release, which in turn hinders transcription elongation and gene expression [45, 49]. We hypothesis that the upregulated R‐loops we observed may affect Pol II pause‐release, which in turn increase the Pol II occupancy in the promoter proximal region. To test this hypothesis, we co‐expressed RNase H together with the σ3, and to examine if RNase H might rescue the upregulated Pol II occupancy induced by σ3. Notably, in the promoter proximal region (TSS±500 bp) of genes *CXCL3* and *RELB*, RNase H expression efficiently reduced the Pol II enrichment compared to σ3 expression alone (Figure 6K–M). This finding further highlighted the functional link between R‐loop accumulation and impaired Pol II pause‐release.

## 3 Discussion

An important mechanism by which viruses counter host immune defenses is by modifying host transcription. vTRs can alter the transcription of hundreds to thousands of host genes [3]. Unraveling the underlying molecular mechanisms by which vTRs alter gene transcription can provide insights into potential anti‐viral strategies. In this study, we found that the REOV outer capsid protein σ3 interacts with the host RNA helicase, DHX9. DHX9 is reported to have both pro‐viral and anti‐viral effects [50]. In the nucleus, DHX9 has an anti‐viral function where it promotes transcription of interferon‐stimulated genes by interacting with STAT1 to recruit RNA‐Pol II to STAT1‐dependent promoters [28]. DHX9 acts analogously at NF‐κB‐dependent promoters, where it interacts with the NF‐κB p65 subunit to recruit RNA pol II to NF‐κB‐dependent genes [30]. We found that through its interaction with DHX9 in the nucleus, σ3 functions as a vTR that counteracts the innate immune response to REOV infection. For TNFα‐induced NF‐κB genes, σ3 impairs the initial recruitment of Pol II by disrupting the interaction between DHX9 and Pol II. Additionally, σ3 impairs the pause‐release of Pol II at the promoter‐proximal sites, thereby repressing the expression of those genes. Conversely, for TNF‐α‐non‐responsive genes, σ3 appears to exert a general repressive effect on transcription by decreasing the Pol II at the promoters through a mechanism that remains to be elucidated.

σ3 is a multifunctional viral protein that functions as part of the outer capsid of the virus and also has important effects in promoting viral replication [24]. We previously showed that σ3 suppresses PKR activation, prevents stress granule formation, and promotes viral replication independently of its capacity to bind dsRNA. The K287T virus, which is unable to bind DHX9, has been implicated in our previous report that it was unable to suppress PKR, synthesized more viral mRNA in the cytoplasm but less viral proteins or negative‐sense viral RNA, and induced abundant cytoplasmic stress granules. These pleiotropic defects may not be rescued by knocking down a single host factor like DHX9, as shown in Figure 3. To clearly dissect the individual function of σ3, an ideal approach would be to use a mutant that specifically disrupts DHX9 binding while retains PKR interaction. Unfortunately, we have been trying to screen such a mutant but failed (data not shown). Future studies, such as structure‐guided mutagenesis or conducting DHX9 depletion in PKR‐deficient cells, would be necessary to fully dissect the distinct roles of this multifunctional protein during REOV replication.

The functions of σ3 parallel those of the Vaccinia virus E3 protein, which also suppresses PKR activation and through its interaction with DHX9 suppresses the transcription of anti‐viral genes [51, 52, 53]. Our current findings help explain how σ3 can rescue the interferon sensitivity of an E3L‐deficient Vaccinia virus [54]. Several previous studies have reported that σ3 localizes to the nucleus in transfected cells [23] and σ3 has been detected in the nucleus of infected cells by immunofluorescence [55]. Our current findings indicate that σ3 likely acts within the nucleus of infected cells to antagonize the transcription of genes involved in the host's innate immune response. σ3 lacks an obvious nuclear localization signal, so an unanswered question is how σ3 is recruited to the nucleus. As the K287T mutant of σ3, which is defective in its interaction with DHX9 is also recruited to the nucleus (data not shown), it is unlikely that σ3 enters the nucleus through direct interaction with DHX9. Infection with some REOV strains leads to inhibition of cellular transcription. Reassortant mapping analysis revealed that the strain differences in the capacity of REOV to inhibit transcription and protein synthesis were associated with the S4 gene segment, which encodes σ3 [56]. Our findings that σ3 ectopic expression increases the cellular levels of R‐loops and decreases overall transcription, suggests that strain differences in the capacity of σ3 to interact with DHX9 may be responsible. Further understanding of how σ3 structurally prevents Pol II from interacting with DHX9 could provide us more insights into this regulation process.

DHX9 is a member of the DEAD‐box/DEAH‐box helicase family and is involved in multiple biological processes, including the regulation of embryonic development, cell proliferation, cancer pathogenesis, inflammation, and innate immunity and immune programming [57]. In the context of viral infection, DHX9 has been shown to have both pro‐ and anti‐viral functions. Several RNA viruses, including Dengue virus [58], Foot‐and‐mouth disease virus [59], and Chikungunya Virus [60] have viral proteins that interact with DHX9 and recruit it to the cytoplasm. A previous study has reported that DHX9 pairs with MAVS in the cytoplasm of REOV‐infected myeloid dendritic cells to sense dsRNA [27]. However, in our current study, in A549 or HEK293T cells, we observed a nuclear translocation of DHX9 upon REOV infection, suggesting a cell‐type specific role for DHX9 during REOV infection. In the nucleus, DHX9 plays important roles in transcriptional regulation and maintaining R‐loop homeostasis. While in vitro experiments suggest that DHX9 can resolve R‐loop structures [61], its regulation of R‐loops in cells is likely more complex. DHX9 inhibits the accumulation of R‐loops at transcription termination sites, thereby promoting transcription termination [47]. However, other studies have found that knockout DHX9 leads to a reduction in overall cellular R‐loop levels. In cells lacking splicesomes, DHX9 can promote R‐loop formation, likely by unwinding RNA secondary structures [62]. The discrepancies between studies highlight the complexity of DHX9's regulation of R‐loops.

σ3 suppresses DHX9 helicase activity in vitro, which is in line with the observed upregulation of cellular R‐loops and the aberrant accumulation of R‐loops at promoter‐proximal regions in virus‐infected cells and cells expressing σ3. How the aberrant accumulation of R‐loops at the promoter‐proximal region relates to transcription output requires further careful investigation. Gene transcription is tightly regulated by multiple rate‐limiting steps, with different transcription factors potentially influencing distinct rate‐limiting steps during transcription. A previous study found that TNF‐α‐stimulated NF‐κB signaling leads to release of paused Pol II from the promoter‐proximal pause site, thereby promoting efficient transcription elongation [44]. Several studies suggest that the excessive R‐loops at the promoter region might affect Pol II pause‐release, which in turn hinders transcription elongation and gene expression [45, 49]. As a result, NF‐κB‐dependent gene expression was significantly downregulated, thereby promoting REOV replication. Through σ3, REOV upregulates the host R‐loop levels to an extent that would affect host gene transcription, thus benefiting its own replication. This is further supported by the fact that overexpression of RNase H in DHX9 deficient cells decreased the viral titer to baseline levels (Figure 7K).

Overall, based on our findings, we propose a model of how σ3 inhibits NF‐κB gene expression: 1) σ3 competes with Pol II for interaction with DHX9, thereby decreasing the DHX9‐dependent recruitment of Pol II to NF‐κB‐dependent promoters. Meanwhile/subsequently, 2) σ3 upregulates the R‐loop levels by inhibiting the helicase activity of DHX9, thereby affecting Pol II pause‐release and ultimately suppressing NF‐κB gene expression (Figure 8H). Our findings highlight the important functions of σ3 as a vTR and describe a new mechanism by which viruses antagonize host innate immune responses.

## 4 Materials and Methods

### 4.1 Cell Culture

Murine L929 cells were purchased from Procell Corporation (Wuhan, China) and were cultured in Minimum Essential Medium (MEM) supplemented with 10% fetal bovine serum (FBS) and 1% penicillin‐streptomycin. HEK293T and A549 were purchased from the American Type Culture Collection (ATCC) (Manassas, VA, USA) and cultured in Dulbecco's modified Eagle's medium (DMEM) supplemented with 10% FBS and 1% penicillin‐streptomycin. All cells were maintained at 37 °C and 5% CO_2._

### 4.2 SDS‐PAGE, Immunoblotting, and Antibodies

Cell pellets were lysed on ice in RIPA buffer (20 mM Tris‐HCl [pH 7.5]; 150 mM NaCl; 1 mM Na_2_EDTA; 1 mM EGTA; 1% [v/v] Nonidet P‐40; 1% sodium deoxycholate; 2.5 mM sodium pyrophosphate; 1 mM β‐glycerophosphate; 1 mM Na_3_VO_4_, 1 µg mL^−1^ leupeptin) for 20 min before being diluted with 5x loading buffer and then boiled at 100 °C for 10 min. Samples were separated by sodium dodecyl sulfate polyacrylamide gel electrophoresis (SDS‐PAGE). After electrophoresis, proteins were transferred to PVDF membrane. Membranes were blocked with 5% milk in TBST (with 0.1% Tween‐20) for 2 h and incubated with the indicated primary antibodies at 4 °C overnight. After incubating with primary antibodies, the membranes were washed with TBST (with 0.1% Tween‐20) and then incubated with HRP‐conjugated secondary antibodies for 2 h at room temperature. Images were collected using Tanon Chemi Dog luminescence imaging system.

All of the antibodies used in this study were summarized in Table S1.

### 4.3 Subcellular Fractionation

Cells were either infected with REO at an MOI of 10 or left untreated. At 6 or 18 h post‐infection, cells were collected and lysed in fractionation buffer (20 mM HEPES [pH 7.4], 10 mM KCl, 2 mM MgCl2, 1 mM EDTA, 1 mM EGTA, 1 mM DTT, and protease inhibitor cocktail). The cell suspension was passed through a 27‐gauge needle 10 times and then incubated on ice for 20 min. The cytoplasmic and nuclear fractions were separated by centrifugation at 3000 rpm for 5 min. Pellets (which contain nuclei) were washed with fractionation buffer, resuspended in nuclei buffer (fractionation buffer supplemented with 10% glycerol and 0.1% SDS) and then sonicated. The supernatants were centrifuged at 8000 rpm for 5 min to obtain clear supernatants, which were cytoplasmic and membrane fractions. Protein concentrations in the nuclear and cytoplasmic fractions were quantified using the BCA assay, and 10 µg of protein per sample was subjected to Western blotting to assess the indicated proteins.

### 4.4 Tandem Affinity Purification and Mass Spectrometry (TAP‐MS)

To identify the σ3 host interactome, InterPlay Mammalian TAP system (Agilent Technologies, Cat No. 240103) was used. Briefly, the coding sequence of σ3 from the T1L REOV strain was introduced into the pNTAP‐A expression vector to create pNTAP‐σ3. Either pNTAP‐A or pNTAP‐σ3 was transfected into 293F suspension cells using TransIT‐293 transfection reagent (Mirus Bio, Cat No. 2706). Cell pellets were collected at 48 h post‐transfection and lysed in lysis buffer that was supplemented with 1x protease inhibitor cocktail (Proteintech, Cat No. PR20032). After three rounds of freeze‐thawing, the cell lysate was clarified by centrifugation at 16 000 g for 10 min. 0.5 M EDTA and 14.4 M β‐mercaptoethanol was added to the clarified supernatant that was then incubated with Streptavidin resin at 4 °C for 2 h. Protein complexes were eluted with SEB buffer. The eluates were added with streptavidin supernatant supplement and then incubated with calmodulin resin at 4 °C for 2 h. After two rounds of washing with CBB buffer, the calmodulin resin was resuspended in 1x SDS loading buffer and boiled for 5 min. Protein complexes were analyzed by LC‐MS/MS. Mass spectrometry data were analyzed by MaxQuant and the results were shown in Data S1.

### 4.5 Coimmunoprecipitation (co‐IP)

Plasmids expressing σ3 (either WT or mutants) or DHX9 mutants were co‐transfected into HEK293T cells at a 1:1 ratio. At 48 h post‐transfection, cells were collected and lysed with NETN buffer (0.5% Nonidet P‐40, 150 mM NaCl, 20 mM Tris‐Cl [pH 8.0], and 0.5 mM EDTA) supplemented with Super nuclease S (Sino Biological, Cat No. SSNP01) or RNase A (Solarbio, Cat No.R1030), or RNase III (Thermo Fisher, Cat No. AM2290) for 20 min at 37 °C. Cell lysates were centrifuged at 12 000 rpm for 10 min at 4 °C and cleared supernatants were transferred into clean tubes before being incubated with anti‐Flag (Smart‐Lifesciences, Cat No. SA109005) or anti‐Myc agarose beads (Beyotime, Cat No.P2285) at 4 °C for 2 h. The agarose beads were washed three times with NETN buffer and subsequently boiled with SDS loading buffer. Samples were then analyzed by immunoblotting.

To examine the interaction between σ3 and DHX9 during REVO infection, cells were infected with REVO at an MOI of 10 and collected at 18 h post‐infection. Cells were lysed with NETN buffer supplemented with Super nuclease S as described above. The lysate was then incubated overnight with DHX9 antibody before being added to pre‐washed Protein A/G beads. After incubation at 4 °C for 2 h, beads were washed and boiled for western blotting analysis.

### 4.6 Expression and Purification of Proteins

Plasmids expressing Flag‐tagged σ3 (wild type or K287T mutant) or Flag‐tagged DHX9 were transfected into 293F suspension cells using polyethylenimine (PEI) (Polysciences, Cat No.24765). At 72 h post‐transfection, cells were harvested and lysed in high‐salt NETN buffer (0.5% Nonidet P‐40, 600 mM NaCl, 20 mM Tris‐Cl [pH 8.0], and 0.5 mM EDTA) supplemented with Super nuclease S (Sino Biological, Cat No. SSNP01). Cell lysates were subjected to two freeze‐thaw cycles before being centrifuged at 10 000 rpm for 30 min. The cleared supernatants were then incubated with anti‐Flag agarose beads for 2 h at 4 °C. After incubation, beads were washed three times with high‐salt NETN buffer. 3x Flag peptides (GenScript, Cat No.RP10586, 150 ng µL^−1^) were used to elute Flag‐tagged proteins. Eluted proteins were then subjected to buffer exchange and concentrated using Amicon Ultra Centrifugal Filters.

His‐tagged GFP‐dRNase H1 was purified as described previously [63]. Briefly, GFP‐tagged dRNase H1 (an enzymatic defective mutant of RNase H1 in which Aspartic acid at position 210 was substituted with Asparagine, and residues 1–27 containing the mitochondrial localization signal were removed) was cloned into the pET28a‐his vector. The plasmid pET28a‐His‐GFP‐dRNase H1 was transformed into *E. Coli* BL21(DE3) and protein was induced by 0.2 mM IPTG at 16°C for 18 h. Bacterial cells were harvested, resuspended in Ni‐binding buffer (50 mM NaH_2_PO_4_, 5 mM Tris‐HCl [pH 7.5], 500 mM NaCl, 0.3% [v/v] Nonidet P‐40, 10% glycerol, 10 mM imidazole, 2 mM β‐mercaptoethanol, 1 mM PMSF, protease cocktail) and lysed by sonication. After centrifugation at 10 000 rpm for 30 min, His‐GFP‐dRNase H1 was purified from the supernatant using Ni‐NTA Beads (Smart‐lifesciences, Cat No. SA004) following the manufacturer's instructions. The purified protein was dialyzed into PBS.

The purity of above proteins was confirmed by Coomassie brilliant blue staining and quantified by BCA assay.

### 4.7 RT‐qPCR

Total RNA was extracted using TransZol up (TransGen, Cat No.Q40831) and then used for reverse transcription using HifairII first Strand cDNA Synthesis SuperMix (Yeasen Cat.11141ES60). After cDNA was generated, the indicated target genes were assessed by Hieff qPCR SYBR Green Master Mix (Yeasen, Cat.11202ES03). All results were normalized to the housekeeping gene *GAPDH*. Primer sequences are listed in Table S2.

### 4.8 Lentivirus Packaging

To produce lentiviruses encoding DHX9‐targeting shRNA or overexpressing RNase H, 6 µg of pLKO‐CMV‐copGFP‐PURO‐shDHX9, or a non‐silencing control pLKO‐CMV‐copGFP‐PURO‐NC (constructed by Tsingke company), or pLVX‐puro‐Flag‐RNase H, together with 3 µg of psPAX2 (Addgene, #12260) and 1.5 µg of pMD2.G (Addgene, #12259), were co‐transfected into HEK293T cells using PEI. At 48 h post‐transfection, the lentivirus‐containing supernatants were collected, filtered through 0.45 µm filters, and then aliquoted and stored at −80 °C until used. Unless otherwise indicated that two individual shRNAs were used, shDHX9‐2 was used in all experiments. Sequences for shRNA targeting DHX9 were listed in Table S2.

### 4.9 Slot Blot

Cellular levels of R‐loops were detected by slot blot as described previously with some modifications [64]. Briefly, after the indicated treatments, A549 cells were washed with cold PBS and then lysed in cell lysis buffer (10% Nonidet P‐40, 2 mM KCl, 0.5 mM PIPES) on ice for 10 min. Nuclei were collected by centrifugation at 500 g for 5 min. The collected nuclei were then lysed in nuclear lysis buffer (1 M Tris‐Cl [pH 8.0], 10% SDS, 0.5 M EDTA) for 10 min on ice. Proteinase K (10 mg mL^−1^) was added and incubated at 55 °C for 6 h. Nucleic acids were extracted using TIANamp Genomic DNA Kit (TIANGEN, Cat No. DP304). Subsequently, 200 ng of nucleic acid from each sample was loaded onto a positively charged nylon membrane using a slot blot apparatus, and then crosslinked using UV (254 nm) for 10 min. Membranes were blocked in 5% milk with TBST (0.1% Tween‐20) for 2 h at RT, and probed with S9.6 antibody overnight at 4 °C and HRP‐conjugated goat anti‐rabbit secondary antibody. Methylene blue staining was performed as a loading control. Band intensities were quantified using ImageJ software.

### 4.10 REOV Replication Curves

REOV replication curves and plaque assays were performed as previously described [24]. Briefly, A549 cells were first infected with lentivirus encoding shRNA targeting DHX9. At 48 h, cells were then infected with either T1L WT or T1L‐σ3 K287T, or T1L‐σ3 R296T, or T1L‐K293T at an MOI of 10. For DHX9 inhibitor assay, A549 cells were first treated with DHX9 inhibitor ATX968 for 2 h, and then infected with indicated REOV. Cells were maintained in medium supplemented with 1 µM DHX9 inhibitor for 24 h. Cells were collected at 0 and 24 h post‐infection. Viral titers were determined by plaque assay.

### 4.11 In Vitro Helicase Assay and Electrophoretic Mobility Shift Assay

Helicase assays were performed as previously described [35]. Sequences of the oligos used for R‐loop construction were listed in Table S2. R‐loops were annealed by mixing equimolar amounts of three oligos in annealing buffer (10 mM Tris–HCl [pH 7.5], 50 mM NaCl), heated to 95 °C for 5 min and then slowly cooled to room temperature. In vitro helicase assays were carried out in unwinding buffer (10 mM Tris–HCl [pH 7.5], 10 mM MgCl_2_, 10% glycerol, 0.2 µg µL^−1^ BSA, 1 mM DTT) with 5 nM R‐loops substrate, 1 mM ATP, 50 mM final concentration of KCl, and the indicated amount of recombinant proteins at 37 °C for 10 min. The reaction was immediately put on ice and being chilled for 5 min before quenched with 10x loading buffer (100 mM Tris–HCl [pH 7.5], 10 mM EDTA, 50% Glycerol, 0.2% SDS, 0.15% Orange G). Samples were then resolved by 11% native PAGE. To quantify the unwinding activity of DHX9, both the band intensity of unwound ssRNA and unresolved R‐loops was quantified using the ImageJ software, and the percent unwinding was plotted. The *K*m of DHX9 was calculated as described previously [65]. A conventional hyperbolic dependence of the reaction rate on substrate concentration was obtained. The plot was generated using the Michaelis‐Menten equation. Nonlinear regression analysis of the plot was used to calculate the *K*m value of DHX9. For the DHX9/σ3 binding assay, the indicated amounts of DHX9 and σ3 were incubated with 5 nM R‐loop substrates in binding buffer (0.5% Nonidet P‐40, 150 mM NaCl, 20 mM Tris‐Cl [pH 8.0], and 0.5 mM EDTA) at 37° C for 15 min. The reaction was then terminated and loaded onto 6% native PAGE gels for 1 h at 100 V. Gels were imaged using a Licor Odessey imaging system under channel 700 nm.

### 4.12 Immunofluorescent (IF) Staining

To assess the subcellular localization of DHX9 in REOV‐infected cells, A549 cells were infected with REO at an MOI of 25. Cells were fixed 18 h post‐infection with 3% paraformaldehyde in buffered PBS at room temperature for 15 min. After washing with 1x PBS, the fixed cells were permeabilized with 0.1% Triton X‐100 in PBS and blocked with 5% FBS in PBS for 30 min. Cells were then co‐stained with rabbit anti‐DHX9 andmouse anti‐σ3 (5C3) (use at 1:1000 dilution) for 1 h and subsequently stained with the corresponding secondary antibodies (Jackson ImmunoResearch, Cat No. 711‐545‐152 and 715‐585‐151) for 1 h. Coverslips were mounted with ProLong Diamond Antifade Mountant (Invitrogen, Cat No.P36961).

To image cellular R‐Loops using purified His‐GFP‐dRNase H1, cells were washed with cold PBS and fixed with ice‐cold methanol for 5 min at −20 °C. For enzymatic digestion, cells were then washed three times with PBS and incubated in 1× RNase H buffer with or without RNase H (New England Biolabs, Cat No.M0297, 1:50 dilution) for 3 h at 37 °C. Following three washes with PBS, cells were blocked with staining buffer (3% BSA in PBS) for 30 min, and then incubated with His‐GFP‐dRNase H1 protein at a final concentration of 3 µg mL^−1^ for 1.5 h at 37 °C. After washing with PBS three times, cells were fixed with 3% PFA for 5 min at room temperature, and then stained with anti‐P65 (1:2000 dilution) for 1 h. The cells were subsequently stained with the corresponding secondary antibodies (Jackson ImmunoResearch, Cat No. 711‐585‐152). Coverslips were mounted with ProLong Diamond Antifade Mountant (Invitrogen, Cat No. P36961).

### 4.13 CUT&Tag and Data Analysis

The CUT&Tag assay was established previously [66] and conducted using Hyperactive Universal the CUT&Tag Assay Kit for Illumina (Vazyme, Cat No. TD903) according to the manufacturer's instructions. Briefly, HEK293 cells were transfected with pcDNA3.1 or pcDNA3.1‐σ3 (either WT or K287T mutant). At 48 h post‐transfection, cells were treated with TNF‐α at 10 ng µL^−1^ for 3 h. Subsequently, approximately 80 000 cells were harvested and washed twice in wash buffer. Concanavalin A beads were then added to the cell suspension and incubated at room temperature for 10 min. Bead‐bound cells were separated from the supernatant and resuspended in antibody buffer. Antibodies against RNA Pol II (Cell Signaling Technology, Cat No.14958, at 1:50 dilution), H3K4Me3 (Abcam, Cat No. ab12209, 1 µg), S9.6 (Absoluteantibody, Cat No. AB01137, with 1 µg), or IgG control (Cell Signaling Technology, Cat No.2729s, with 1 µg) were added to the cells, and incubated at 4 °C overnight, for S9.6 CUT & Tag, 10 µg µL^−1^ of RNase A was supplemented as negative control. The following day, the secondary antibody diluted in dig‐wash buffer was added to the beads and incubated at room temperature for 1 h. After incubation, beads were washed three times with dig‐wash buffer. pA/G‐Tnp transposomes diluted in dig‐300 buffer were incubated with beads at room temperature for 1 h. After three washes with dig‐300 buffer, samples were then tagmented with TTBL from the kit. After 1 h of tagmentation, proteinase K and spike‐in were added. Fragmented DNA was purified using DNA Extract Beads. The eluted DNA was amplified using Illumina i7/i5 indexed primers. After that, VAHTS DNA Clean Beads (Vazyme #N411) were employed for the purification of the libraries, which were then subjected to quality control and Illumina sequencing.

The CUT&Tag reads were checked for quality and trimmed using Fastp. Reads were aligned to the Ensembl hg38 human reference genome using Bowtie2 (version 2.4.4) with the options “–very‐sensitive –no‐mixed –no‐discordant –phred33 ‐I 10 ‐X 700 ‐p 12”. Duplicate reads were removed using Picard tools for downstream analyses. Samtools was used to convert the read format and sort the reads. To visualize the genomic occupancy from the transcription start site (TSS) to the transcription end site (TES), bam files were converted into BigWig files using the bamCoverage function from deepTools, with the ‘normalizeUsing RPKM’ parameter set. Subsequently, computeMatrix and plotHeatmap from deeptools were used with the options “–beforeRegionStartLength 3000 –regionBodyLength 5000 –afterRegionStartLength 3000 –skipZeros ‐o”. Then, SEACR was employed for peak scanning across the entire genome with the option of “0.05 non‐stringent”. ChIPseeker was used to annotate the positions of peaks within genomic features. DEseq2 was used for differential analysis.

To quantitatively compare the CUT & Tag signals within the promoters of TNF‐α‐responsive genes under different conditions, we first extracted the promoter regions of TNF‐α‐responsive genes from the GTF file of the hg38 human reference genome. Promoter regions were defined as 500 bp upstream and downstream of the TSS. Reads numbers in the promoter regions were counted by ‘bedtools coverage’ and then normalized to counts per million (CPM) based on the total reads of the corresponding sample. RNA Pol II pausing index (PI) was determined using a reported method with modification [45, 46]. Briefly, PI was defined as the ratio between normalized Pol II read density on TSS flanking region (−300 to +300 bp) against its signal in gene body (+1 to +3000 bp). Paired *t*‐tests were used to evaluate the signal differences within the promoters of TNF‐α‐responsive genes under different conditions.

### 4.14 Chromatin Immunoprecipitation (ChIP)‐qPCR

A ChIP assay was performed using the BeyoChIP Enzymatic ChIP Assay kit (Beyotime, Cat No. P2083) and carried out according to the manufacturer's instructions. Antibodies used in the ChIP assay are: Histone H3K4me3 (at 2.4 µg mL^−1^), S9.6 (at 2.4 µg mL^−1^), and RNA pol II (at 2.4 µg mL^−1^). Anti‐rabbit IgG was used as a negative control. qPCR was performed to assess the DNA enrichment using Hieff qPCR SYBR Green Master Mix (Yeasen, Cat.11202ES03). The primers used in ChIP‐qPCR assay were listed in Table S2.

### 4.15 Statistical Analysis and Replicates

The results were expressed as the means ± SD of at least three independent experiments, unless stated otherwise. Each imaging experiment involved at least five different scanning areas. Comparisons of means between two groups were conducted by two‐tailed Student's t test. For comparisons involving more than two groups, statistical significance was determined using ANOVA followed by a Tukey post hoc analysis. Statistical calculations were carried out and visualized by GraphPad Prism 8 (GraphPad). *P* values were marked by asterisks. A *p*‐value of less than 0.05 was regarded as statistically significant (n.s., not significant; *p < 0.05, **p < 0.01, ***p < 0.001, and ****p < 0.001).

## Funding

National Natural Science Foundation of China (32200133 to Y.G and 82241065 to D.Z) National Institute of Allergy and Infectious Diseases (AI121216 to J.S.L.P). The funders have no role in study design, data collection and analysis.

## Conflicts of Interest

The authors declare no conflicts of interest.

## Supporting information




**Supporting File**: advs73480‐sup‐0001‐SuppMat.docx.

## Data Availability

The CUT & Tag sequencing data in this study have been deposited in the Gene Expression Omnibus (GEO) database under accession number: GSE290339 and GSE310004. The RNA‐seq data in this study have been deposited in the GEO database under accession number: GSE290340. Data for mass spectrometry proteomic data are available via ProteomeXchange with identifier PXD061062.

## References

[1] C. Citu , L. Chang , A. M. Manuel , N. Enduru , and Z. Zhao , “Identification and Catalog of Viral Transcriptional Regulators in Human Diseases,” Iscience 28 (2025): 112081. 40124487 10.1016/j.isci.2025.112081 PMC11928865

[2] I. Nečasová , M. Stojaspal , E. Motyčáková , T. Brom , T. Janovič , and C. Hofr , “Transcriptional Regulators of Human Oncoviruses: Structural and Functional Implications for Anticancer Therapy,” NAR Cancer 4 (2022): zcac005. 35252867 10.1093/narcan/zcac005 PMC8892037

[3] X. Liu , T. Hong , S. Parameswaran , et al., “Human Virus Transcriptional Regulators,” Cell 182 (2020): 24–37. 32649876 10.1016/j.cell.2020.06.023 PMC7346790

[4] J. S. Y. Ho , Z. Zhu , and I. Marazzi , “Unconventional Viral Gene Expression Mechanisms as Therapeutic Targets,” Nature 593 (2021): 362–371. 34012080 10.1038/s41586-021-03511-5

[5] S. Ramasubramanyan , A. Kanhere , K. Osborn , K. Flower , R. G. Jenner , and A. J. Sinclair , “Genome‐Wide Analyses of Zta Binding to the Epstein‐Barr Virus Genome Reveals Interactions in both Early and Late Lytic Cycles and an Epigenetic Switch Leading to an Altered Binding Profile,” Journal of Virology 86 (2012): 12494–12502. 23015699 10.1128/JVI.01705-12 PMC3497672

[6] D. Portal , H. Zhou , B. Zhao , et al., “Epstein–Barr Virus Nuclear Antigen Leader Protein Localizes to Promoters and Enhancers With Cell Transcription Factors and EBNA2,” Proceedings of the National Academy of Sciences 110 (2013): 18537–18542. 10.1073/pnas.1317608110 PMC3832032 24167291

[7] J. E. Reeder , Y.‐T. Kwak , R. P. McNamara , C. V. Forst , and I. Orso , “HIV Tat Controls RNA Polymerase II and the Epigenetic Landscape to Transcriptionally Reprogram Target Immune Cells,” Elife 4 (2015): 08955. 10.7554/eLife.08955 PMC4733046 26488441

[8] D.‐L. Zheng , L. Zhang , N. Cheng , et al., “Epigenetic Modification Induced by Hepatitis B Virus X Protein via Interaction With De Novo DNA Methyltransferase DNMT3A,” Journal of Hepatology 50 (2009): 377–387. 19070387 10.1016/j.jhep.2008.10.019

[9] C. H. Ludwig , A. R. Thurm , D. W. Morgens , et al., “High‐Throughput Discovery and Characterization of Viral Transcriptional Effectors in Human Cells,” Cell Systems 14 (2023): 482–500. 37348463 10.1016/j.cels.2023.05.008 PMC10350249

[10] K. M. Lelli , M. Slattery , and R. S. Mann , “Disentangling the Many Layers of Eukaryotic Transcriptional Regulation,” Annual Review of Genetics 46 (2012): 43–68. 10.1146/annurev-genet-110711-155437 PMC4295906 22934649

[11] P. Cramer , “Organization and Regulation of Gene Transcription,” Nature 573 (2019): 45–54. 31462772 10.1038/s41586-019-1517-4

[12] T. García‐Muse and A. R. L. Aguilera , “R Loops: From Physiological to Pathological Roles,” Cell 179 (2019): 604–618. 31607512 10.1016/j.cell.2019.08.055

[13] M. P. Crossley , M. Bocek , and K. A. Cimprich , “R‐Loops as Cellular Regulators and Genomic Threats,” Molecular Cell 73 (2019): 398–411. 30735654 10.1016/j.molcel.2019.01.024 PMC6402819

[14] L. A. Sanz , S. R. Hartono , Y. W. Lim , et al., “Prevalent, Dynamic, and Conserved R‐Loop Structures Associate With Specific Epigenomic Signatures in Mammals,” Molecular Cell 63 (2016): 167–178. 27373332 10.1016/j.molcel.2016.05.032 PMC4955522

[15] K. Wang , H. Wang , C. Li , et al., “Genomic Profiling of Native R Loops With a DNA‐RNA Hybrid Recognition Sensor,” Science Advances 7 (2021): abe3516. 10.1126/sciadv.abe3516 PMC7888926 33597247

[16] P. A. Ginno , P. L. Lott , H. C. Christensen , I. Korf , and F. Chédin , “R‐Loop Formation Is a Distinctive Characteristic of Unmethylated Human CpG Island Promoters,” Molecular Cell 45 (2012): 814–825. 22387027 10.1016/j.molcel.2012.01.017 PMC3319272

[17] P. B. Chen , H. V. Chen , D. Acharya , O. J. Rando , and T. G. Fazzio , “R Loops Regulate Promoter‐Proximal Chromatin Architecture and Cellular Differentiation,” Nature Structural & Molecular Biology 22 (2015): 999–1007. 10.1038/nsmb.3122 PMC4677832 26551076

[18] L. Müller , R. Berkeley , T. Barr , and E. Ilett , “Past, Present and Future of Oncolytic Reovirus,” Cancers 12 (2020): 3219. 33142841 10.3390/cancers12113219 PMC7693452

[19] R. Bouziat , R. Hinterleitner , J. J. Brown , et al., “Reovirus Infection Triggers Inflammatory Responses to Dietary Antigens and Development of Celiac Disease,” Science 356 (2017): 44–50. 28386004 10.1126/science.aah5298 PMC5506690

[20] Q. Zhang , M. J. Lenardo , and D. Baltimore , “30 Years of NF‐κB: a Blossoming of Relevance to Human Pathobiology,” Cell 168 (2017): 37–57. 28086098 10.1016/j.cell.2016.12.012 PMC5268070

[21] A. J. McNamara , A. D. Brooks , and P. Danthi , “The Reovirus Σ3 Protein Inhibits NF‐κB‐Dependent Antiviral Signaling,” Journal of Virology 96 (2022): 0051522. 10.1128/jvi.00515-22 PMC9093121 35416720

[22] A. J. McNamara and P. Danthi , “Loss of IKK Subunits Limits NF‐κB Signaling in Reovirus‐Infected Cells,” Journal of Virology 94 (2020): 00382. 10.1128/JVI.00382-20 PMC7199397 32161168

[23] Z. Yue and A. J. R. Shatkin , “Regulated, Stable Expression and Nuclear Presence of Reovirus Double‐Stranded RNA‐Binding Protein Sigma3 in HeLa Cells,” Journal of Virology 70 (1996): 3497–3501. 8648682 10.1128/jvi.70.6.3497-3501.1996 PMC190223

[24] Y. Guo , M. M. Hinchman , M. Lewandrowski , et al., “The Multi‐Functional Reovirus Σ3 Protein Is a Virulence Factor That Suppresses Stress Granule Formation and Is Associated With Myocardial Injury,” PLOS Pathogens 17 (2021): 1009494. 10.1371/journal.ppat.1009494 PMC8291629 34237110

[25] T. Lee and J. Pelletier , “The Biology of DHX9 and Its Potential as a Therapeutic Target,” Oncotarget 7 (2016): 42716–42739. 27034008 10.18632/oncotarget.8446 PMC5173168

[26] R. Ullah , J. Li , P. Fang , S. Xiao , and L. Fang , “DEAD/H‐Box Helicases:Anti‐Viral and Pro‐Viral Roles During Infections,” Virus Research 309 (2022): 198658. 34929216 10.1016/j.virusres.2021.198658

[27] Z. Zhang , B. Yuan , N. Lu , V. Facchinetti , and Y.‐J. Liu , “DHX9 Pairs With IPS‐1 To Sense Double‐Stranded RNA in Myeloid Dendritic Cells,” The Journal of Immunology 187 (2011): 4501–4508. 21957149 10.4049/jimmunol.1101307 PMC3656476

[28] X. Ren , D. Wang , G. Zhang , et al., “Nucleic DHX9 Cooperates With STAT1 to Transcribe Interferon‐Stimulated Genes,” Science Advances 9 (2023): add5005. 10.1126/sciadv.add5005 PMC9897671 36735791

[29] S. Leone , D. Bär , C. F. Slabber , D. Dalcher , and R. Santoro , “The RNA Helicase DHX9 Establishes Nucleolar Heterochromatin, and this Activity Is Required for Embryonic Stem Cell Differentiation,” EMBO Reports 18 (2017): 1248–1262. 28588071 10.15252/embr.201744330 PMC5494521

[30] Y. C. Ng , W.‐C. Chung , H.‐R. Kang , et al., “A DNA‐Sensing–Independent Role of a Nuclear RNA Helicase, DHX9, in Stimulation of NF‐κB–Mediated Innate Immunity Against DNA Virus Infection,” Nucleic Acids Research 46 (2018): 9011–9026. 30137501 10.1093/nar/gky742 PMC6158622

[31] J. J. Knowlton , I. Fernández de Castro , A. W. Ashbrook , et al., “The TRiC Chaperonin Controls Reovirus Replication Through Outer‐Capsid Folding,” Nature Microbiology 3 (2018): 481–493. 10.1038/s41564-018-0122-x PMC5874176 29531365

[32] P. Schütz , E. Wahlberg , T. Karlberg , et al., “Crystal Structure of Human RNA Helicase A (DHX9): Structural Basis for Unselective Nucleotide Base Binding in a DEAD‐Box Variant Protein,” Journal of Molecular Biology 400 (2010): 768–782. 20510246 10.1016/j.jmb.2010.05.046

[33] W. Yuan , Q. Al‐Hadid , Z. Wang , et al., “TDRD3 Promotes DHX9 Chromatin Recruitment and R‐Loop Resolution,” Nucleic Acids Research 49 (2021): 8573–8591. 34329467 10.1093/nar/gkab642 PMC8421139

[34] M. H. Daniels , J. Castro , Y.‐T. Lee , et al., “Discovery of ATX968: an Orally Available Allosteric Inhibitor of DHX9,” Journal of Medicinal Chemistry 68 (2025): 9537–9554. 40298172 10.1021/acs.jmedchem.5c00252 PMC12067447

[35] A. Dutta , Y. Kwon , and P. Sung , “Biochemical Analysis of RNA‐DNA Hybrid and R‐Loop Unwinding via Motor Proteins,” Methods in Molecular Biology 2528 (2022): 305–316. 35704200 10.1007/978-1-0716-2477-7_20

[36] S. J. Boguslawski , D. E. Smith , M. A. Michalak , et al., “Characterization of Monoclonal Antibody to DNA · RNA and Its Application to Immunodetection of Hybrids,” Journal of Immunological Methods 89 (1986): 123–130. 2422282 10.1016/0022-1759(86)90040-2

[37] C. Bou‐Nader , A. Bothra , D. N. Garboczi , S. H. Leppla , and J. Zhang , “Structural Basis of R‐Loop Recognition by the S9.6 Monoclonal Antibody,” Nature Communications 13 (2022): 1641. 10.1038/s41467-022-29187-7 PMC8960830 35347133

[38] S. Liu , L. He , J. Wu , et al., “DHX9 Contributes to the Malignant Phenotypes of Colorectal Cancer via Activating NF‐κB Signaling Pathway,” Cellular and Molecular Life Sciences 78 (2021): 8261–8281. 34773477 10.1007/s00018-021-04013-3 PMC11072136

[39] H. Kwak , N. J. Fuda , L. J. Core , and J. T. Lis , “Precise Maps of RNA Polymerase Reveal How Promoters Direct Initiation and Pausing,” Science 339 (2013): 950–953. 23430654 10.1126/science.1229386 PMC3974810

[40] M. Zhao , P. Chauhan , C. A. Sherman , et al., “NF‐κB Subunits Direct Kinetically Distinct Transcriptional Cascades in Antigen Receptor‐Activated B Cells,” Nature Immunology 24 (2023): 1552–1564. 37524800 10.1038/s41590-023-01561-7 PMC10457194

[41] Q. J. Cheng , S. Ohta , K. M. Sheu , et al., “NF‐κB Dynamics Determine the Stimulus Specificity of Epigenomic Reprogramming in Macrophages,” Science 372 (2021): 1349–1353. 34140389 10.1126/science.abc0269 PMC8489855

[42] A. Adelaja , B. Taylor , K. M. Sheu , Y. Liu , S. Luecke , and A. Hoffmann , “Six Distinct NFκB Signaling Codons Convey Discrete Information to Distinguish Stimuli and Enable Appropriate Macrophage Responses,” Immunity 54 (2021): 916–930. 33979588 10.1016/j.immuni.2021.04.011 PMC8184127

[43] A. Oeckinghaus and S. Ghosh , “The NF‐ B Family of Transcription Factors and Its Regulation,” Cold Spring Harbor Perspectives in Biology 1 (2009): a000034. 20066092 10.1101/cshperspect.a000034 PMC2773619

[44] C. G. Danko , N. Hah , X. Luo , et al., “Signaling Pathways Differentially Affect RNA Polymerase II Initiation, Pausing, and Elongation Rate in Cells,” Molecular Cell 50 (2013): 212–222. 23523369 10.1016/j.molcel.2013.02.015 PMC3640649

[45] R. R. Ng , Z. Lin , Y. Zhang , et al., “R‐Loop Resolution by ARIP4 Helicase Promotes Androgen‐Mediated Transcription Induction,” Science Advances 10 (2024): adm9577. 10.1126/sciadv.adm9577 PMC11259169 39028815

[46] D. S. Day , B. Zhang , S. M. Stevens , et al., “Comprehensive Analysis of Promoter‐Proximal RNA Polymerase II Pausing Across Mammalian Cell Types,” Genome Biology 17 (2016): 120. 27259512 10.1186/s13059-016-0984-2 PMC4893286

[47] A. Cristini , M. Groh , M. S. Kristiansen , and N. Gromak , “RNA/DNA Hybrid Interactome Identifies DXH9 as a Molecular Player in Transcriptional Termination and R‐Loop‐Associated DNA Damage,” Cell Reports 23 (2018): 1891–1905. 29742442 10.1016/j.celrep.2018.04.025 PMC5976580

[48] H. Wang , Z. Fan , P. V. Shliaha , et al., “H3K4me3 Regulates RNA Polymerase II Promoter‐Proximal Pause‐Release,” Nature 615 (2023): 339–348. 36859550 10.1038/s41586-023-05780-8 PMC9995272

[49] B. P. Belotserkovskii , J. H. Soo Shin , and P. C. Hanawalt , “Strong Transcription Blockage Mediated by R‐Loop Formation Within a G‐Rich Homopurine–Homopyrimidine Sequence Localized in the Vicinity of the Promoter,” Nucleic Acids Research 45 (2017): 6589–6599. 28498974 10.1093/nar/gkx403 PMC5499740

[50] B. Bonaventure and C. Goujon , “DExH/D‐Box Helicases at the Frontline of Intrinsic and Innate Immunity Against Viral Infections,” Journal of General Virology 103 (2022): 103. 10.1099/jgv.0.001766 36006669

[51] H. W. Chang , J. C. Watson , and B. L. Jacobs , “The E3L Gene of Vaccinia Virus Encodes an Inhibitor of the Interferon‐Induced, Double‐Stranded RNA‐Dependent Protein Kinase,” Proceedings of the National Academy of Sciences 89 (1992): 4825–4829. 10.1073/pnas.89.11.4825 PMC49180 1350676

[52] A. Dempsey , S. E. Keating , M. Carty , and A. G. Bowie , “Poxviral Protein E3–Altered Cytokine Production Reveals that DExD/H‐Box Helicase 9 Controls Toll‐Like Receptor–Stimulated Immune Responses,” Journal of Biological Chemistry 293 (2018): 14989–15001. 30111593 10.1074/jbc.RA118.005089 PMC6166711

[53] K. J. Dueck , Y. S. Hu , P. Chen , et al., “Mutational Analysis of Vaccinia Virus E3 Protein: the Biological Functions Do Not Correlate With Its Biochemical Capacity to Bind Double‐Stranded RNA,” Journal of Virology 89 (2015): 5382–5394. 25740987 10.1128/JVI.03288-14 PMC4442530

[54] E. Beattie , K. L. Denzler , J. Tartaglia , M. E. Perkus , E. Paoletti , and B. L. Jacobs , “Reversal of the Interferon‐Sensitive Phenotype of a Vaccinia Virus Lacking E3L by Expression of the Reovirus S4 Gene,” Journal of Virology 69 (1995): 499–505. 7527085 10.1128/jvi.69.1.499-505.1995 PMC188598

[55] S. Schmechel , M. Chute , P. Skinner , R. Anderson , and L. Schiff , “Preferential Translation of Reovirus mRNA by a σ3‐Dependent Mechanism,” Virology 232 (1997): 62–73. 9185589 10.1006/viro.1997.8531

[56] A. Mohamed , J. R. Smiley , and M. Shmulevitz , “Polymorphisms in the Most Oncolytic Reovirus Strain Confer Enhanced Cell Attachment, Transcription, and Single‐Step Replication Kinetics,” Journal of Virology 94 (2020): 01937. 10.1128/JVI.01937-19 PMC6997772 31776267

[57] C. F. Bourgeois , F. Mortreux , and D. Auboeuf , “The Multiple Functions of RNA Helicases as Drivers and Regulators of Gene Expression,” Nature Reviews Molecular Cell Biology 17 (2016): 426–438. 27251421 10.1038/nrm.2016.50

[58] Y. Wang , X. Chen , J. Xie , et al., “RNA Helicase A Is an Important Host Factor Involved in Dengue Virus Replication,” Journal of Virology 93 (2019): 01306. 10.1128/JVI.01306-18 PMC6363994 30463971

[59] P. Lawrence and E. Rieder , “Identification of RNA Helicase A as a New Host Factor in the Replication Cycle of Foot‐and‐Mouth Disease Virus,” Journal of Virology 83 (2009): 11356–11366. 19710149 10.1128/JVI.02677-08 PMC2772801

[60] R. Matkovic , E. Bernard , S. Fontanel , et al., “The Host DHX9 DExH‐Box Helicase Is Recruited to Chikungunya Virus Replication Complexes for Optimal Genomic RNA Translation,” Journal of Virology 93 (2019): 01764. 10.1128/JVI.01764-18 PMC6364007 30463980

[61] P. Chakraborty and F. Grosse , “Human DHX9 Helicase Preferentially Unwinds RNA‐Containing Displacement Loops (R‐Loops) and G‐Quadruplexes,” DNA Repair 10 (2011): 654–665. 21561811 10.1016/j.dnarep.2011.04.013

[62] P. Chakraborty , J. T. J. Huang , and K. Hiom , “DHX9 Helicase Promotes R‐Loop Formation in Cells With Impaired RNA Splicing,” Nature Communications 9 (2018): 4346. 10.1038/s41467-018-06677-1 PMC6195550 30341290

[63] M. P. Crossley , J. R. Brickner , C. Song , et al., “Catalytically Inactive, Purified RNase H1: A Specific and Sensitive Probe for RNA–DNA Hybrid Imaging,” Journal of Cell Biology 220 (2021): 202101092. 10.1083/jcb.202101092 PMC8266564 34232287

[64] P. Ramirez , R. J. Crouch , V. G. Cheung , and C. Grunseich , “R‐Loop Analysis by Dot‐Blot,” Journal of Visualized Experiments 22 (2021): 62069. 10.3791/62069 PMC8506650 33554969

[65] M. Ahmad , A. Ansari , M. Tarique , A. T. Satsangi , and R. Tuteja , “Plasmodium Falciparum UvrD Helicase Translocates in 3′ to 5′ Direction, Colocalizes With MLH and Modulates Its Activity Through Physical Interaction,” PLoS One 7 (2012): 49385. 10.1371/journal.pone.0049385 PMC3503981 23185322

[66] H. S. Kaya‐Okur , D. H. Janssens , J. G. Henikoff , K. Ahmad , and S. Henikoff , “Efficient Low‐Cost Chromatin Profiling With CUT&Tag,” Nature Protocols 15 (2020): 3264–3283. 32913232 10.1038/s41596-020-0373-x PMC8318778

