# Supplementary material for: A dsRNA Viral Transcriptional Regulator Evades Innate Immunity by Hijacking Host CoTranscription Factor DHX9
Source: Adv Sci (Weinh). 2025 Dec 22;13(10):e12262. doi: 10.1002/advs.202512262 (PMC12915084; doi:10.1002/advs.202512262)
Supplement: Supplementary file 1 — Supporting File: advs73480‐sup‐0001‐SuppMat.docx. [file ADVS-13-e12262-s001.docx]

Supplementary Materials for

**A dsRNA Viral Transcriptional Regulator Evades Innate Immunity by Hijacking Host Co-Transcription Factor DHX9.**

Xueyang Pang *et al.*

* Corresponding authors: [John](mailto:jsp7@cornell.edu(John) S. L. Parker (jsp7@cornell.edu); Yingying Guo ([yingyingguo0228@tmu.edu.cn](mailto:yingyingguo0228@tmu.edu.cn)); Dongming Zhou ([zhoudongming@tmu.edu.cn](mailto:zhoudongming@tmu.edu.cn))

**This file includes:**

Figs. S1 to S7

Tables S1 to S2

Data S1 to S3


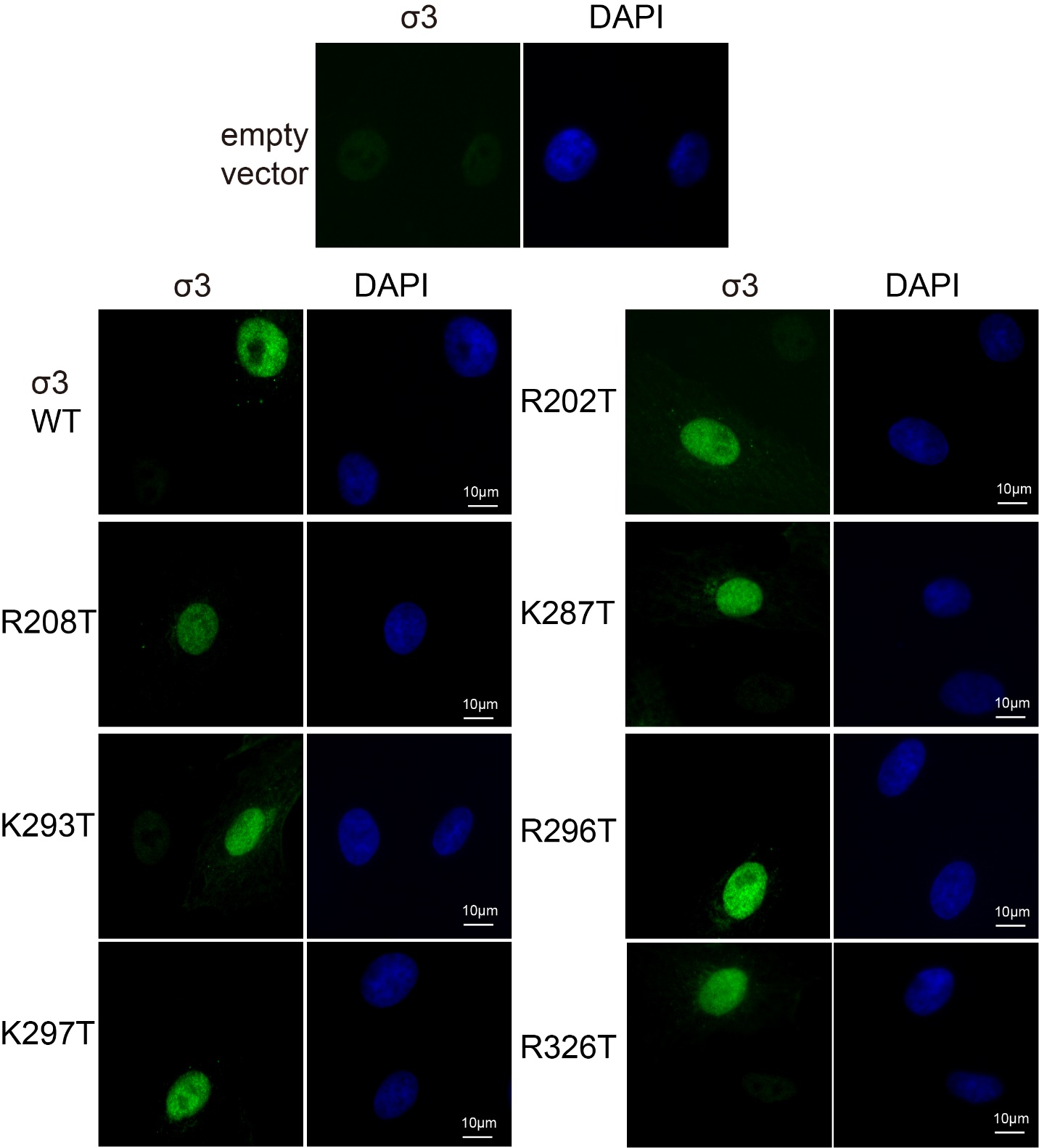


**Fig. S1. The localization patterns of all σ3 mutants were similar to that of the wild-type protein.**

A549 cells were transfected with Flag-tagged σ3 (WT and various mutants). At 48 hrs post transfection, cells were fixed and immunofluorescence staining was performed using a polyclonal antibody against σ3 (green) and DAPI (blue).


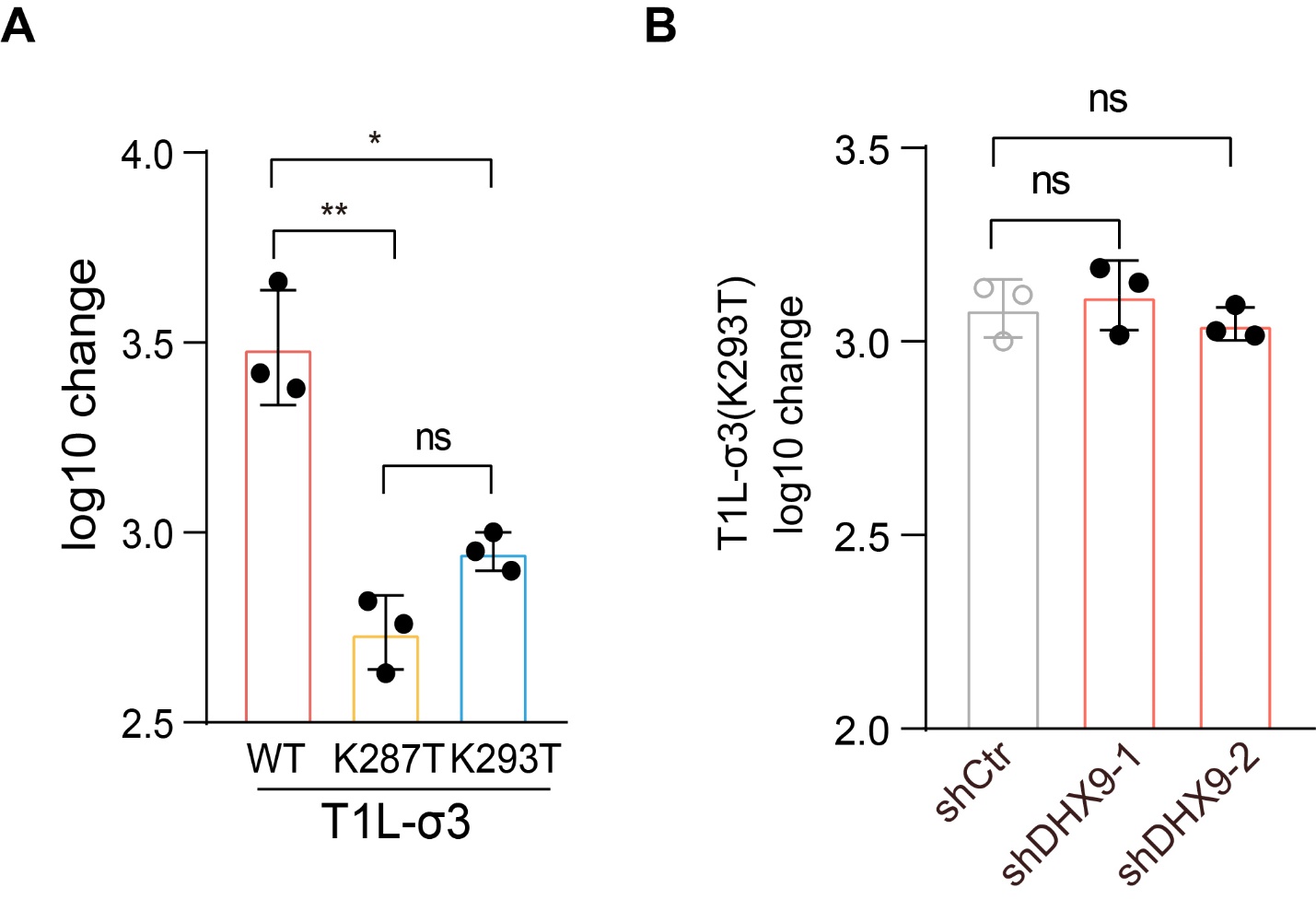


**Fig. S2. Viral growth of T1L-K293T**

(A)Compared to WT virus, K287T and K293T virus exhibited a replication defect. Viral growth was shown as changes in viral titer from 0 to 24 h pi. Data shown represent the mean ± s.d. of three independent experiments.

(B)Viral growth of T1L-K293T was assessed in DHX9-deficient A549 cells. A549 cells were first infected with lentivirus encoding shRNA against DHX9. At 48 hours post-transduction, cells were infected with T1L-K293T at MOI 10. Viral growth was shown as changes in viral titer from 0 to 24 h pi. Paired *t*-tests was used to analyze the differences (ns=no significant, * P < 0.05, ** P < 0.01, *** P < 0.001).


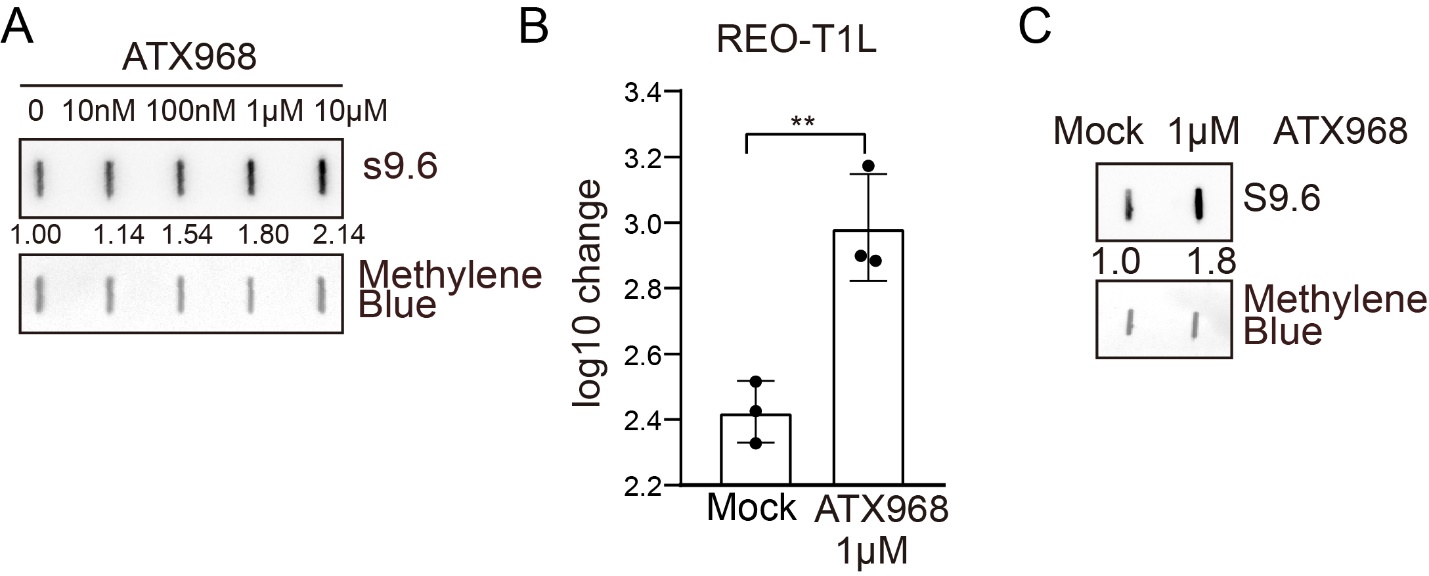


**Fig. S3. DHX9 restricts REOV replication via its helicase activity.**

(A) Cellular R-loop levels were upregulated upon treatment with the DHX9 inhibitor ATX968. A549 cells were treated with increasing concentration of ATX968 for 2 hrs. Genomic DNA were then extracted, and R-loop levels were assessed by slot blot assay using the S9.6 antibody.

(B) Cells were pre-treated with 1µM ATX968 for 2 hrs, followed by infection with REOV at an MOI of 10. The cells were maintained in medium supplemented with 1µM DHX9 inhibitor for 24 hrs. Viral growth was evaluated as changes in viral titer from 0 to 24 h pi. Paired t-tests was used to analyze the differences (ns=no significant, * P < 0.05, ** P < 0.01, *** P < 0.001). (C) The efficient of DHX9 inhibition was verified by slot blot analysis.


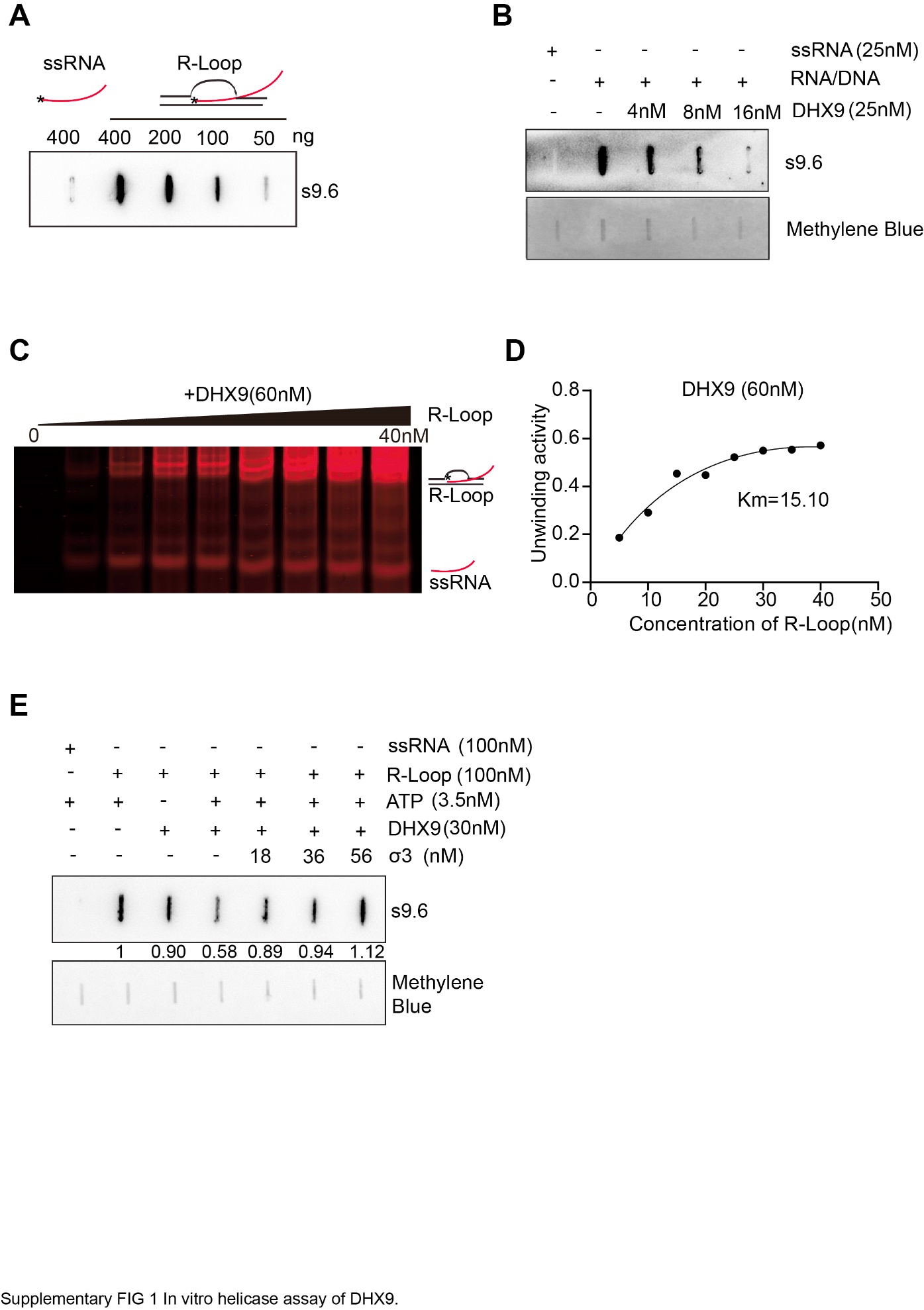


**Fig. S4. *In vitro* helicase assay of DHX9.**

(A) The R-loop substrates were verified by slot blot with S9.6 antibody. (B) DHX9 resolves R-loops in vitro. The in vitro helicase assay on R-loops was performed by incubating the R-loop substrates with increasing amounts of recombinant DHX9 at 37°C for 10 mins, and subsequently analyzed by native polyacrylamide gel electrophoresis and fluorescence imaging. (C and D) Kinetics of DHX9 helicase activity. *K*_m_ of helicase activity was calculated from the plot as described in methods and materials section. (E) σ3 inhibits the helicase activity of DHX9. DHX9 was incubated with increasing amounts of σ3 at room temperature for 30 min, and then subjected for helicase assay by adding R-loop substrates and ATP.


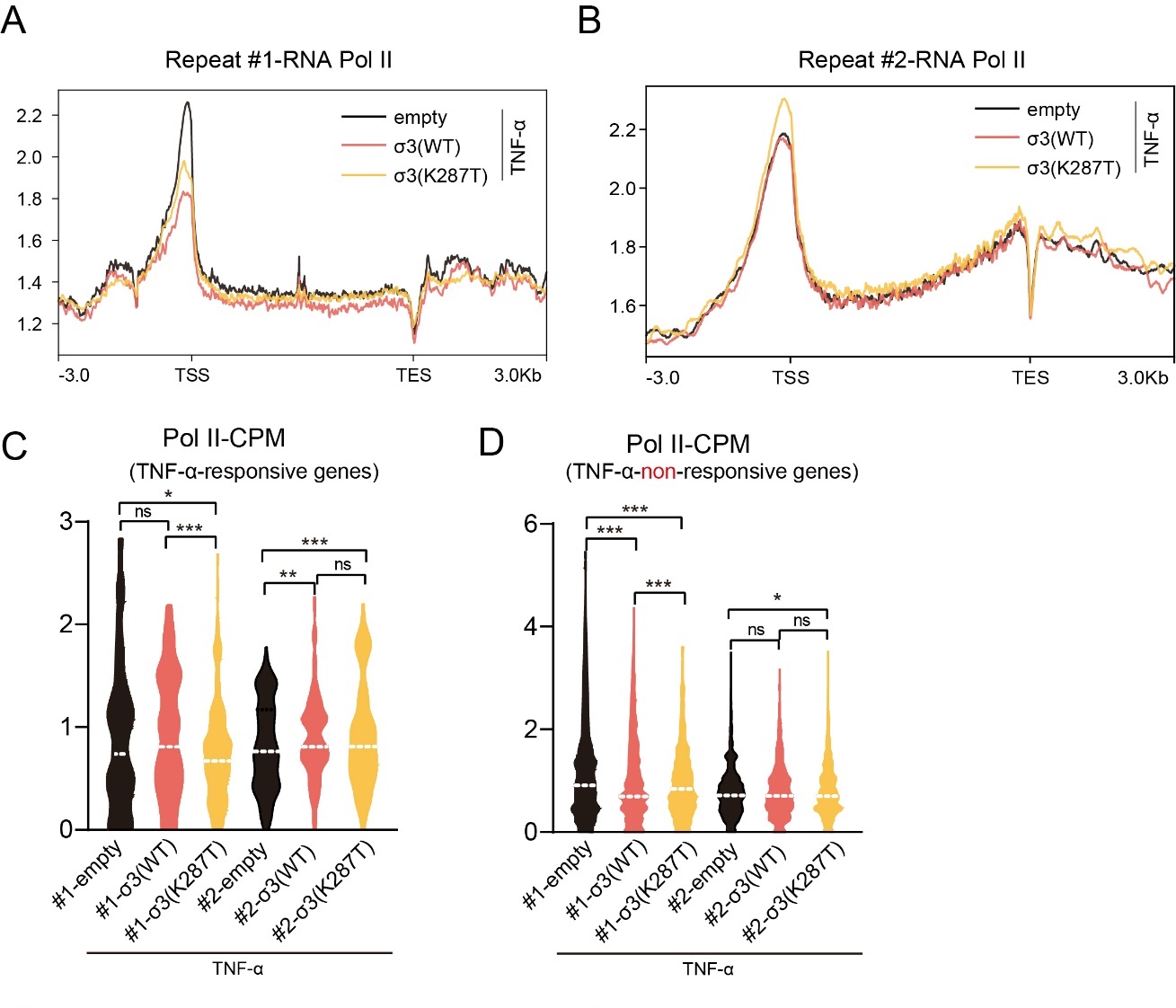


**Fig. S5. Pol II CUT&Tag Signal Profiles and Counts Per Million (CPM) for Two Biological Replicates.**

(A and B) Average Pol II CUT & Tag signal profile across genic regions (from 3 kb upstream of the TSS to 3 kb downstream of the TES) in cells expressing σ3 or K287T cells following TNF-α treatment for two biological replicates. Panel (A) shows biological replicate 1, and panel (B) shows biological replicate 2.

(C and D) Pol II occupancy, measured in Counts per Million (CPM) at the promoter proximal region (TSS ± 500 bp) is displayed for two biological replicates. Statistical significance was assessed using paired *t*-test (ns, not significant; * P < 0.05, ** P < 0.01, *** P < 0.001).


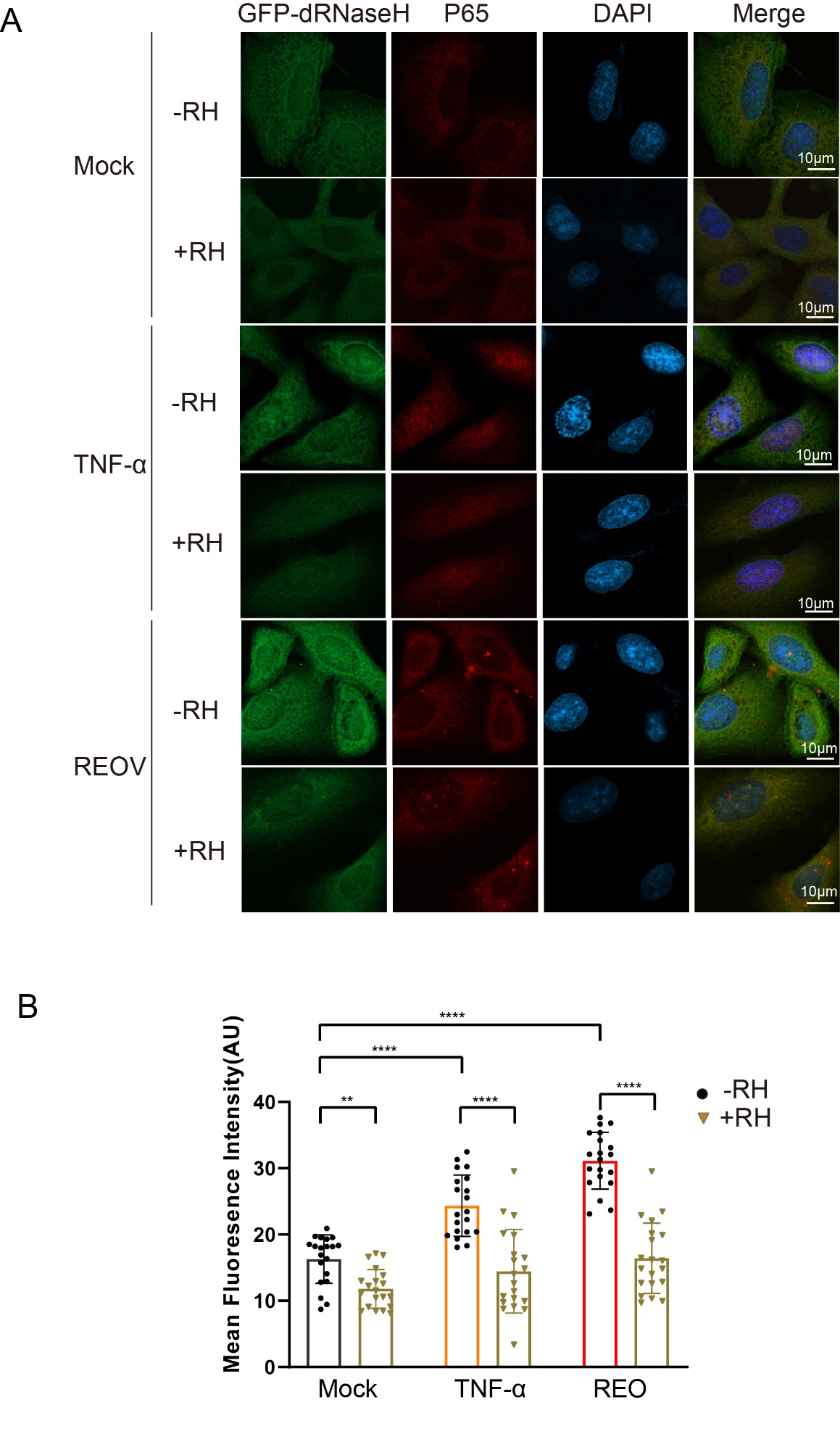


**Fig. S6. Imaging of cellular R-loop by GFP-dRNase H.**

(A) A549 cells were treated with TNF-α (10 ng/ml) for 3 hrs or infected with REOV at MOI 20 for 6 hrs. Cells were then fixed in ice-cold methanal for 5 min in -20°C. After fixation, cells were treated with or without RNase H for 3 h at 37°C. After washing with PBS, cells were blocked with staining buffer and then incubated with His-GFP-dRNase H1 protein at a final concentration of 3 µg/ml for 1.5 h at 37°C. Following the incubation, cells were stained with anti-P65 (1:2000 dilution) for 1 h and corresponding secondary antibody. (B) Quantification of mean nuclear GFP-dRNH1 intensities for the conditions shown in (A). At least 20 nuclei were scored per condition. Non paired *t* test was used to analyze the differences (** *P* < 0.01, **** *P*<0.0001).

**
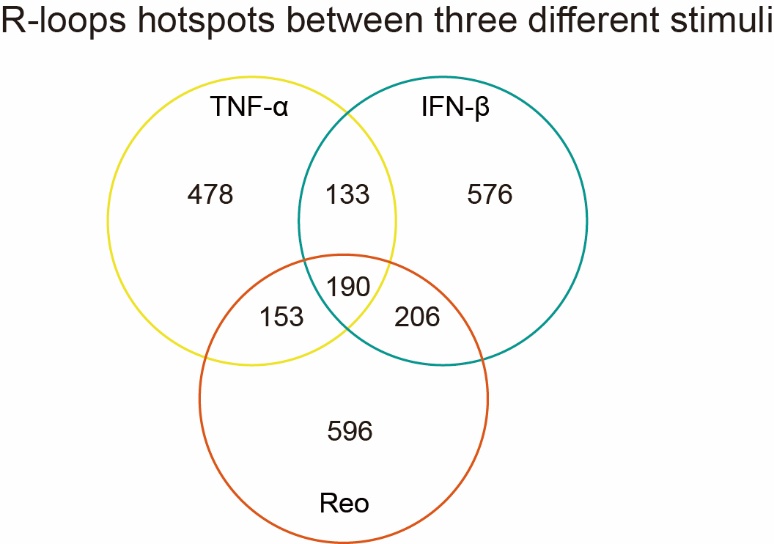
**

**Fig. S7. R-loops hotspots between three different stimuli.**

Venn diagram illustrates the overlapping peaks of R-loops identified in cells treated with TNF-α (yellow), IFN-β (blue), or Reo(red).

**Table S1. Commercial antibodies used in this study**

| **Antibody** | **Type** | **Supplier** | **Cat.No.** |
| --- | --- | --- | --- |
| Flag | Mouse | Sigma | F1804 |
| DHX9 (for WB, IP) | Rabbit | Abcam | ab26271 |
| DHX9 (for IF) | Rabbit | Abcam | ab183731 |
| Myc | Mouse | Cell Signaling Technology | 2276s |
| GAPDH | HRP conjugated | Proteintech | HRP-60004 |
| β-actin | HRP conjugated | Proteintech | HRP-60008 |
| RNA Pol II (for WB) | Mouse | Santa cruz | sc-55492 |
| RNA Pol II (for Cut &Tag) | Rabbit | Cell Signaling Technology | 14958 |
| RNA Pol II (for ChIP-qPCR) | Mouse | Cell Signaling Technology | 2629 |
| H3K4me3 | Rabbit | Abcam | ab12209 |
| IgG | Rabbit | Cell Signaling Technology | 2729 |
| S9.6 | Rabbit | Absolute antibody | AB01137 |
| p65 | Rabbit | Cell Signaling Technology | 8242 |
| TIAR | Mouse | Santa cruz | sc-398372 |
| goat anti-rabbit secondary antibody | HRP conjugated | Abcam | AB6721 |
| goat anti-mouse secondary antibody | HRP conjugated | Abcam | AB6789 |

**Table S2. Oligonucleotide sequences used in this study**

Primers for RT-qPCR

| GENE | Forward (5’-3’) | Reverse (5’-3’) |
| --- | --- | --- |
| *RELB* | TGTGGTGAGGATCTGCTTCCAG | TCGGCAAATCCGCAGCTCTGAT |
| *CXCL2* | AGAGGCTGAGACTAACCCAGA | TTTCATGCTGGAGGCGAGAG |
| *NFKBIA* | TCCACTCCATCCTGAAGGCTAC | CAAGGACACCAAAAGCTCCACG |
| *UBE2Z* | GATGACAACGGGCAATAACACA | CATCAGGGACTGGATAGAGATGA |
| *IL5* | GGAATAGGCACACTGGAGAGTC | CTCTCCGTCTTTCTTCTCCACAC |
| *GAPDH* | GTCTCCTCTGACTTCAACAGCG | ACCACCCTGTTGCTGTAGCCAA |

| GENE | Forward (5’-3’) | Reverse (5’-3’) |
| --- | --- | --- |
| *ACTIN-TSS±500bp* | CCGCTGGGTTTTATAGGGCG | TCCTCAATCTCGCTCTCGCT |
| *ACTIN-TSS-1kb* | TGTGGACATCTCTTGGGCAC | AGGGCAGTTGCTCTGAAGTC |
| *ACTIN-gene body* | CTCATGGCCTTGTCACACGA | GCCGTTTTCCGTAGGACTCT |
| *RELB-TSS±500bp* | CGAGACCTTGGAGGTTTCCGA | AGGCCTGCTTAGAGCCATCA |
| *RELB-TSS-1kb* | GGTCTGAGAGCGCCTGGCAT | CATCCTGAGCTGTGTGCCTA |
| *RELB-gene body* | TTTAATGCCAGCAGGGCTGA | TTTTTGCTCAGCAGCCACAG |
| *CXCL3-TSS±500bp* | CGGAGCTCCAGATCGATCCG | GCTGTATCTTCAGCGAGGTG |
| *CXCL3-TSS-1kb* | AAACAGCTGCCTGCTGATGA | CCCCCAGTGCTGAATACCAG |
| *CXCL3-gene body* | GCTGCACACAACCCTATGAA | CCCTAATGGGCTTCAGACCT |

Primers for ChIP-qPCR

shRNAs used for knockdown

| shRNA | sequence (5’-3’) |
| --- | --- |
| *shDHX9-1* | ACGACAATGGAAGCGGATATA |
| *shDHX9-2* | GGGCTATATCCATCGAAATTT |
| *shCTR* | GGTTCTCCGAACGTGTCACGT |

Oligonucleotide used *in vitro* helicase assay and EMSA

| Name | sequence (5’-3’) |
| --- | --- |
| *ssDNA-1* | CATTGCATATTTAAAACATGTTGGATCCCACGTTGCATGCTGATAGCCTACTAGAGCTGTATGAATTCAAATGACCTCTTATCAAGTGAC |
| *ssDNA-2* | GTCACTTGATAAGAGGTCATTTGAATTCATGGCTTAGAGCTTAATTGCTGAATCTGGTGCTGGGATCCAACATGTTTTAAATATGCAATG |
| *RNA* | Cy5.5-GCACCAGAUUCAGCAAUUAAGCUCUAAGCCGCUGACGGCUCGAUG  CUGAUCGUAGCAUCG |

**Data S1(separate File)**

**σ3 interactome from TAP-MS.xlsx**

**Data S2(separate File)**

**List of genes that are silenced by σ3**

**Data S3(separate File)**

**RNA-seq analysis for TNFa-responsive genes and TNFa-non-responsive genes.xlsx**
